# Redefining *GBA* gene structure unveils the ability of Cap-independent, IRES-dependent gene regulation

**DOI:** 10.1038/s42003-022-03577-5

**Published:** 2022-07-13

**Authors:** Keiko Miyoshi, Hiroko Hagita, Taigo Horiguchi, Ayako Tanimura, Takafumi Noma

**Affiliations:** 1 Department of Oral Bioscience, Tokushima University Graduate School of Biomedical Sciences, Tokushima, 770-8504 Japan; 2 Division of Food & Health Sciences, Department of Environmental and Symbiotic Sciences, Faculty of Environmental and Symbiotic Sciences, Prefectural University of Kumamoto, Kumamoto, 862-8502 Japan; 3 Department of Nutrition and Health Promotion, Faculty of Human Life Studies, Hiroshima Jogakuin University, 4-13-1 Ushita-higashi, Higashi-ku, Hiroshima, 732-0063 Japan

**Keywords:** Gene expression, Transcriptional regulatory elements

## Abstract

Glucosylceramide is the primary molecule of glycosphingolipids, and its metabolic regulation is crucial for life. Defects in the catabolizing enzyme, glucocerebrosidase (GCase), cause a lysosomal storage disorder known as Gaucher disease. However, the genetic regulation of GCase has not been fully understood. Here we show the redefined structure of the GCase coding gene (*GBA*), and clarify the regulatory mechanisms of its transcription and translation. First, alternative uses of the two *GBA* gene promoters were identified in fibroblasts and HL60-derived macrophages. Intriguingly, both *GBA* transcripts and GCase activities were induced in macrophages but not in neutrophils. Second, we observed cap-independent translation occurs via unique internal ribosome entry site activities in first promoter-driven *GBA* transcripts. Third, the reciprocal expression was observed in *GBA* and miR22-3p versus *GBAP1* transcripts before and after HL60-induced macrophage differentiation. Nevertheless, these findings clearly demonstrate novel cell-type-specific *GBA* gene expression regulatory mechanisms, providing new insights into GCase biology.

## Abstract (web summary)

The cell type-specific expression of the glucocerebrosidase gene, associated with the lysosomal storage disorder called Gaucher disease, is linked to cis- and trans-regulatory transcriptional and translational mechanisms.

## Introduction

Sphingolipids are critical components of the cell membrane, whose metabolites are bioactive lipids linked with many biological functions, such as endocytosis, cell cycle progression, apoptosis, cell senescence, cell survival, migration, and inflammation^1,2^. Subsequently, ceramide can be phosphorylated to ceramide-1-phosphate and glycosylated to generate glucosylceramide (GlcCer) or galactosylceramide^3^. Alternatively, GlcCer is a fundamental molecule in ninety percent of mammalian glycosphingolipids, essential for cellular homeostasis, growth, and development^3,4^.

GlcCer is hydrolyzed to generate glucose and ceramide using three distinct glucosylceramidases encoded by *GBA1* (hereafter *GBA*), *GBA2*, and *GBA3*^3^. Among them, the *GBA* gene encodes glucosylceramidase beta (EC 3.2.1.45, hereafter GCase), GCase is a lysosomal hydrolase (optimum pH: 4–5) localized in the lysosomal membrane and ubiquitously expressed in the body. Importantly, Gaucher disease (GD), a lysosomal storage disorder, is caused by a defective GCase due to more than 500 recessive *GBA* mutations. Hence, most known GD patients are compound heterozygotes^5^. In these patients, pathophysiological GD phenotypes are frequently observed in the specific systems/tissues, such as the reticuloendothelial system, the skeletal system, and the brain, due to the affected cells which accumulate the substrate GlcCer are the monocyte/macrophage lineage including microglial brain cells, called Gaucher cells^6^.

Therefore, it is essential to understand the cell-type-specific and metabolic role of GCase, elucidating the structural basis for *GBA* gene regulation. To date, three groups have reported basic information on the gene structure of *GBA*^7–9^. The first group reported the commonly used sequence as a reference. As described, this gene is approximately 7.6 kb in size, is composed of 11 exons, and localizes at chromosome 1q21^7,8^. Also, the *GBA* pseudogene (*GBAP1*) exists at the 16 kb region downstream of *GBA*, approximately 5.7 kb in size^7,8^. Thus, *GBA* and *GBAP1* sequences are 96% identical and exhibit similar exon-intron structural organizations^7,8^. Alternatively, the second group demonstrated that the *GBA* gene in the human genome comprises 13 exons, two promoters, and five alternative splicing variants^9^. Finally, the third group reported a database that showed the existence of two promoters, 12 exons, and five alternative splicing variants, different from those in the second group’s report (Gene ID 2629). Furthermore, another database showed that *GBAP1*, a highly homologous pseudogene of *GBA*, was located approximately 12 kb downstream of *GBA*. Thus, the *GBA* gene structure is confusing and poorly defined.

Therefore, we report a more redefined organization of the *GBA* gene structure in this study. We also discussed additional cell-type-specific regulatory mechanisms of *GBA* gene expression, including the presence of two alternative promoters and cap-independent translational machinery via internal ribosome entry site (IRES) activities in the first promoter. Additionally, *GBAP1* might be involved in the regulation of *GBA* expression, a different role from competitive endogenous RNA (ceRNA)^10,11^. Hence, our findings shed light on the cell-type-specific regulation of GlcCer’s metabolism.

## Results

### Determination of the 5′ end of *GBA*

*GBA* structures have been reported to have various 5′ ends and exon numbering schemes^7–9^. However, some discrepant criteria are causing complexities. Therefore, to understand the regulation of *GBA* gene expression, we first identified the transcription start site(s) (TSS(s)) of the *GBA* gene. For this study, we performed the RNA ligase-mediated rapid amplification of cDNA ends (RLM-RACE) analysis using total mRNA isolated from human dermal fibroblasts (DFs). The sequence with accession number J03059 was used as a reference for *GBA*^7^. Subsequently, to avoid amplification of the *GBAP1* cDNA, the 1st *GBA*-specific primer was designed using a *GBA-*specific region (55 bp) at exon 9 of J03059 (that is not present in the *GBAP1* sequence; J03060). Furthermore, a nested *GBA*-specific primer was designed at exon 5 (Supplementary Fig. 1a). Then, amplified cDNA fragments containing the 5’ ends were sequenced, after which their identities and positions were determined in comparison with the reference genome. We observed three additional variants containing a new exon and confirmed that this new exon was not derived from *GBAP1* in any cDNAs (Supplementary Fig. 1b–f).

**Fig. 1:**
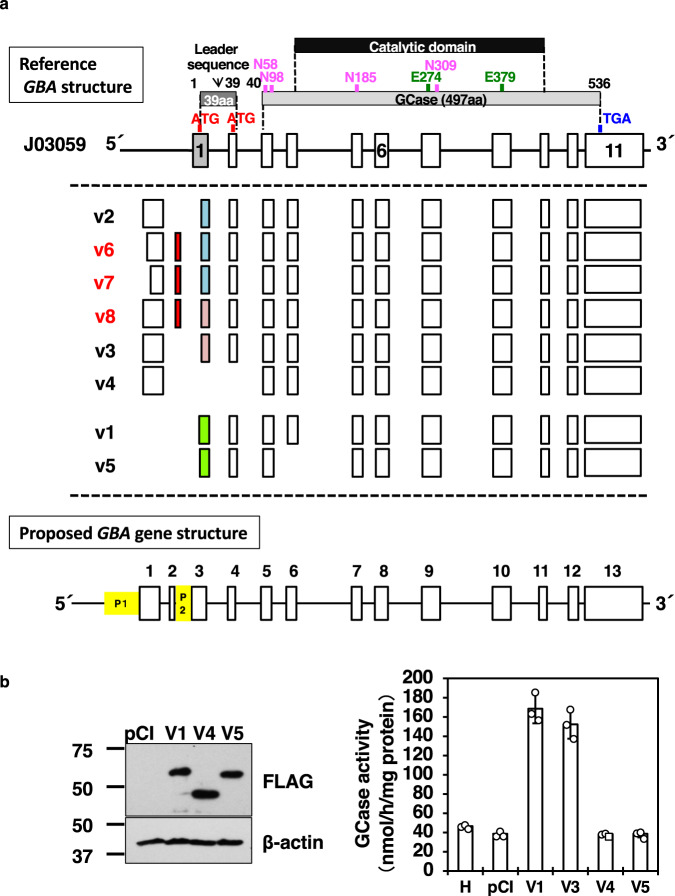
Determination of the 5’ ends of a *GBA* gene. **a** Summary cartoon of the 5’ ends of all *GBA* variants. Upper: Exon-intron structures of the reference sequence J03059 and the corresponding translated GCase protein region (gray box) with a catalytic domain (black box). “ATG” in red, the start codon, “TGA” in blue, the stop codon, “N” in pink, glycosylation sites; “E” in green, catalytic residues. Middle (within dotted lines): Alignment of the eight variants (vs) with adjusted exon positions compared to J03059. Variant accession numbers are shown in the data availability section. As shown, variants are categorized into two groups: P1-driven variants (v2, v6, v7, v8, v3, and v4) and P2-driven variants (v1 and v5). Red boxes indicate newly identified exon two. Exon three subdomains are shown in colored boxes (3i, gray; 3ii, green; 3iii, blue; 3iv, pink). Lower: The proposed *GBA* gene structure. Yellow boxes indicate the P1 and P2 promoter regions. The number above each box indicates the redefined exon number. **b** GCase protein expression and enzymatic activities of variants v4 and v5. (Left) The GCase protein expression was analyzed using western blot analysis. Representative data is shown. pCI, an empty vector-transfected HEK293 cell lysate; V1, V4, V5, each variant overexpressed HEK293 cell lysate. (Right) GCase activity of each variant transfected HEK293 cells. H, control HEK293 cells (no treatment); V3, variant v3 in pCIneo-transfected cells; others, similar to the right panel. *n* = 3 biologically independent samples. Data represent the mean ± STDEV.

Figure 1a shows a cartoon summarizing the *GBA* gene structure based on our findings, which are as follows: (1) the *GBA* gene has two promoters, P1 (distal) and P2 (proximal), with multiple TSSs and splicing variants; (2) the structure of the reference *GBA* gene J03059^8^ is based on the transcript beginning at the P2 promoter; (3) the P1 promoter regulates several alternative splicing variants, such as v2, v3, and v4 (accession numbers: v2, NM_001005741; v3, NM_001005742; v4, NM_001171811), including novel variants v6, v7, and v8 identified in this study, containing a newly identified exon that has been deposited in the database (accession numbers: v6, LC050340; v7, LC050341; v8, LC050342); (4) the P2 promoter controls two variants, v1 and v5 (accession numbers: v1, NM_000157; v5, NM_001171812), in addition to the reference transcript J03059; and (5) unique alternative splicing patterns are present in variants v4 and v5. Additionally, we observed that variant v4 was transcribed beginning at the P1 promoter, and that the first exon was connected to the proposed exon five. Moreover, variant v5 was transcribed beginning at the P2 promoter. However, the proposed exon six was skipped.

Additionally, our study showed that sequences we obtained for variants v4 and v5 differed from those reported by Svobodova et al. (cited as v4, NM_001005749.1; v5, NM_001005750.1)^9^. However, these sequences had previously been removed from GenBank due to the absence of transcripts and proteins. Therefore, to confirm whether the newly identified splice variants v4 and v5 encoded any functional protein, we employed cDNA expression by transfecting the synthesized v4 and v5 coding sequences in an expression vector into HEK293 cells. As shown in Fig. 1b, we could detect both v4 and v5 proteins without the enzymatic activity.

This study also redefined the *GBA* gene structure and organization with a new numbering of two promoters and 13 exons (Fig. 1a). Notably, a fine-scale analysis of the RLM-RACE-produced cDNA revealed four additional splicing acceptor sites in exon 3 (indicated by subdomains i, ii, iii, and iv near the 5′ end of exon 3 in Supplementary Fig. 1g). Additionally, we observed that although differences between the previously reported J03059 and variant v1 were due to the alternative usage of either exon 3i or 3ii, differences between variants v2 and v3 were due to differentially using either exon 3iii or 3iv. Besides, three new variants were observed: v6, v7, and v8, which were produced due to multiple TSSs. Results also detected that the alternative splicing of exon two from exon one (redefined in this study) was under the P1 promoter’s control. Intriguingly, *GBAP1* had previously been reported to have a similar diversity of alternative splicing patterns, including three splicing acceptor sites at its third exon^10^.

**Fig. 2:**
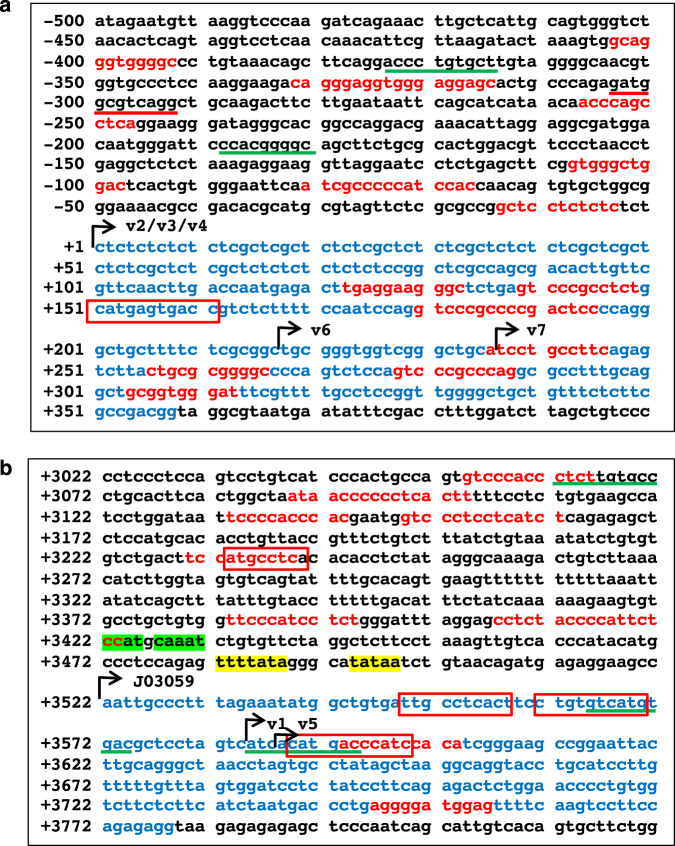
TSSs of *GBA* variants in the P1 and P2 promoter regions. Panels (**a**, **b**) indicate P1 and P2 promoter regions spanning 500 bp with the following exon sequences, respectively. The most upstream TSS from P1 promoter is designated as +1, and the following sequence positions are indicated to the right side. Arrows, individual TSSs of the variants (v); blue letters, exon sequences; red letters, SP1 binding sites; yellow highlights, TATA box; green highlights, CAAT box; red boxes, AP1 (JUN/FOS) binding sites; red underlines, CREB binding sites; green underlines, TFEB binding sites.

We further inspected the upstream sequences of each promoter. Although several Sp1 binding sites were found in both the P1 and P2 promoters, putative TATA boxes were identified at the P2 promoter but not in the P1 promoter (Fig. 2a, b). However, structural analysis of the RACE cDNA clearly revealed that *GBA* gene expression was intricately regulated by combining alternative promoter usage and alternative splicing, resulting in the great diversity of 5′ untranslated regions (UTRs) in *GBA* transcripts.

**Fig. 3:**
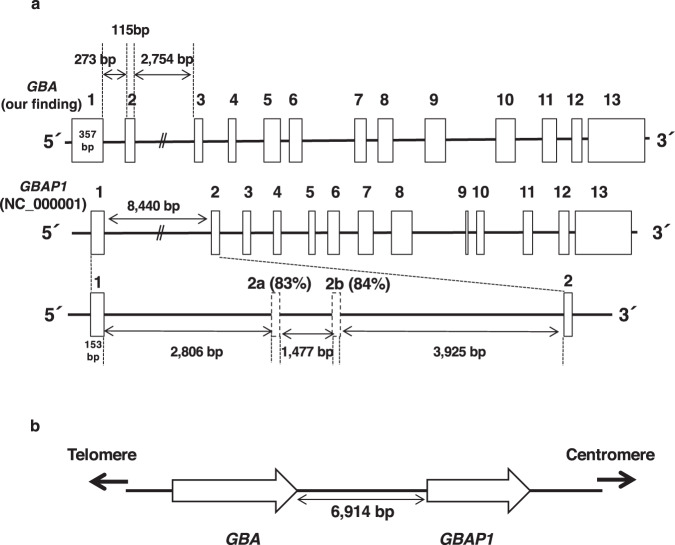
Gene structure and chromosomal position of *GBA* and *GBAP1*. **a** Structural comparison of *GBA* and *GBAP1*. The white boxes indicated exons. Based on the exon 1 and 2 sequences of *GBA*, upstream exons of *GBAP1* were predicted through a homology search of the genome database and are depicted with dashed-line boxes. **b** The positional relationship between *GBA* and *GBAP1* in chromosome 1. White arrows indicate the direction of *GBA* and *GBAP1*.

It has been reported that *GBA* and *GBAP1* share 96% identical sequences with the same exon-intron organization^7,8^. Thus, we subsequently analyzed the structure of the *GBAP1* gene to confirm its structural similarity. As shown in Fig. 3a, the *GBAP1* gene in the database (NC_000001) contained 13 exons, whereas exon one and exon two were 8440 bp apart. A homology search, including the novel exon 2 of *GBA*, also revealed two highly homologous regions in *GBAP1*, exon 2a and 2b (117 bp and 118 bp, respectively). These exons were approximately 2.8 kb and 4.3 kb downstream of exon 1. Furthermore, comparing exon 2a and exon 2b sequences with the new exon 2 of *GBA* revealed an 83% and 84% similarity, respectively. We also recalculated the distance between the *GBA* and *GBAP1* genes and obtained an expanse of 6914 bp, suggesting an evolutionary gene duplication, which had previously been reported^12^ (Fig. 3b).

### Characterization of variant *GBA* transcripts

To confirm whether the two promoters were active and transcribed all variants, we subsequently analyzed expression profiles of *GBA* variants in two fibroblasts: DFs and oral fibroblasts (OFs), through quantitative PCR (qPCR). Previously, we had reported that both DFs and OFs expressed glycolipid metabolism-related genes stronger than OF-derived iPS cells^13^. However, we also detected a unique feature of OFs, committed to the cranial neural crest cell-lineage with plasticity and longevity^13^. Therefore, we analyzed *GBA* expression in both fibroblasts.

**Fig. 4:**
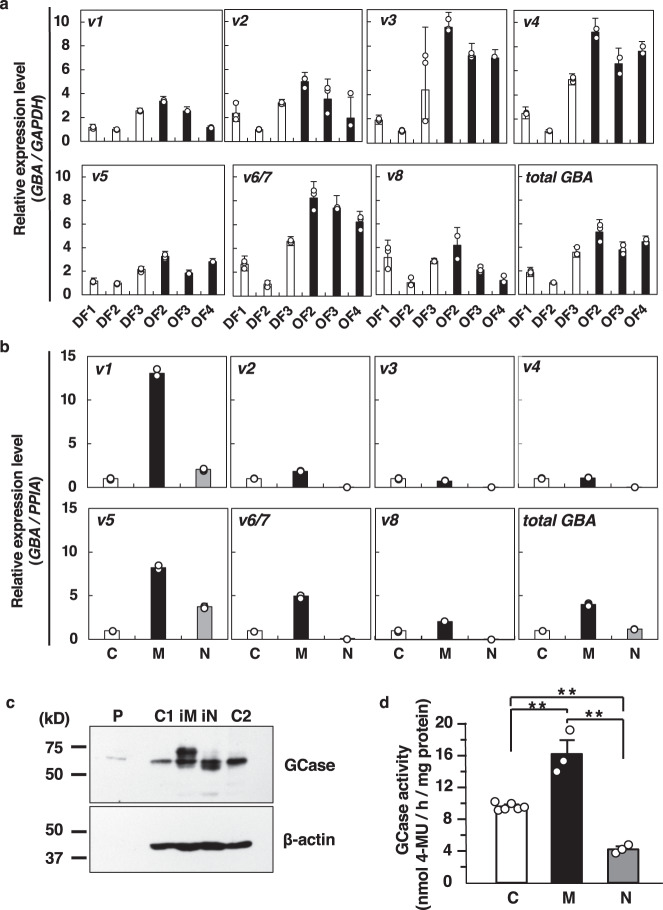
Cell type-specific expression of *GBA* variants. **a** Comparison of the expression levels of *GBA* transcripts in fibroblasts. The expression levels of *GBA* transcripts were normalized to those of *GAPDH*, and expressed as the relative quantity to that of DF2. Each experiment was performed in triplicate. DF, dermal fibroblasts; OF, oral mucosa fibroblasts. **b** Expression levels of *GBA* transcripts among different cell types using an in vitro hematopoietic differentiation system of HL60 cells. The expression levels of *GBA* were normalized to those of *PPIA*, and expressed as the relative quantity to that of control HL60 cells. Each experiment was performed in triplicate except *v6/7*, N in *v3*, C in *total GBA* were duplicate. C, control HL60 cells (no treatment); M, iMacs; N, iNeuts. **c** The expression levels of GCase protein by western blot analysis. Representative western blot analysis data is shown. C1 and C2, two individual controls (nontreated HL60 cells); M, iMacs; N, iNeuts, P, positive control (human *GBA* in pCIneo-transfected HEK293 cell lysate). **d** GCase activity. C, control HL60 cells (no treatment); M, iMacs; N, iNeuts. Each experiment was performed in triplicate except control (control, *n* = 6). **p < 0.01. Data represent the mean ± STDEV.

We detected both P1 and P2 promoter-driven variants in DFs and OFs (Fig. 4a). Interestingly, expression levels of P1 promoter-driven variants: v3, v4, and v6/7, were higher in OFs than those in DFs. However, P2 promoter-driven variants (v1 and v5) were expressed at similar levels in DFs and OFs. Moreover, total GBA expression levels were slightly higher in OFs than DFs. Therefore, results showed that although the P1 promoter was major in OFs, both P1 and P2 promoters were active, and all variants were transcribed in both fibroblasts.

Next, because GlcCer accumulates in Gaucher cells which are derived from the macrophages of GD patients^5^, we wondered whether the unique regulation of *GBA* gene expression was observed in macrophages. Hence, we applied an in vitro hematopoietic cell differentiation-inducing system using HL60 cells^14–16^ (Supplementary Fig. 4b).

Surprisingly, the P2 promoter-driven variants v1 and v5 were more highly expressed in phorbol 12-myristate 13-acetate (PMA)-induced macrophages (iMacs) than P1 promoter-driven variants v2, and v4, major transcripts in OFs (Fig. 4b). However, levels in all-*trans* retinoic acid (ATRA)-induced neutrophils (iNeuts) were less than half of those observed in iMacs. These findings suggested that the P2 promoter of the *GBA* gene was specifically activated during the iMac differentiation of HL60 cells. Subsequently, we inspected both promoter sequences to confirm whether the P2 promoter activation was specific in iMacs or through PMA effects. As shown in a previous report^17^, three 12-O-tetradecanoylphorbol-13-acetate (TPA) response elements (TREs, *TGAGTCAG*; also known as activator protein 1 (AP1) binding sites) in the P2 promoter region existed (Fig. 2b, red boxes). However, although the P1 promoter contained no TREs, one TRE was located within exon 1 (Fig. 2a, red boxes). Additionally, a cAMP response element-binding protein (CREB) attachment site was identified in the P1 promoter but not in the P2 promoter (Fig. 2a, b, red underline). These data suggested the P2 promoter as a direct target of PMA for selecting specific promoter usage.

Interestingly, a CLEAR element, the binding site for transcription factor EB (TFEB), was detected in both promoter sequences (Fig. 2a, b, green underlines). TFEB is a known *GBA* gene regulator^18^. Subsequently, although *TFEB* mRNA expression levels were detected 1.6-fold higher in iMacs, the levels were 50% lower in iNeuts than parental HL60 cells (Supplementary Fig. 2c).

We also confirmed whether a functional GCase protein was produced in iMacs and iNeuts. Interestingly, although GCase protein expression was strongly detected with sifted-up signal in iMacs, it was a slightly shifted-down band in iNeuts compared to untreated HL60 cells (Fig. 4c). Of note, GCase levels in untreated HL60 were observed, but they had some variation. Nevertheless, GCase activity was enhanced only 1.6-fold in iMac cells and 0.5-fold in iNeuts compared to untreated HL60 cells (Fig. 4d). These results suggested discrepancies between *GBA* mRNA expression levels and the GCase protein, as have been already reported^8,19^. Yet, their regulatory mechanisms remain unknown.

### Cap dependency of translational control

Long 5’ UTRs have been recognized as platforms of translational control through RNA ultrastructures or modifications with molecular interactions^20^. Therefore, since our sequence analysis of the *GBA* gene revealed an unusually long 5’ UTR (Table 1), it prompted us to investigate the translational regulation of the *GBA* gene.

**Table 1:** Sequence properties of the 5’ UTRs of *GBA* transcripts.

Variant	Promoter	5’ UTR length (nt)	GC%^b^	∆*G*^c^
J03059	P2	232	48.28	−68.6
v1	P2	169	49.11	−47.3
v2	P1	428	60.51	−132.3
v3	P1	409	61.12	−131.4
v4	P1	506	61.46	−159.9
v5	P2	166	49.40	−47.3
v6	P1	350	58.57	−129.3
v7	P1	331	57.70	−120.5
v8^a^	P1	547	59.60	−184.7

^a^v8 is the predicted sequence combined with v3.

^b^GC content was calculated by GENETYX-Mac network ver. 19.0.0.

^c^∆*G* (free energy) was analyzed by the IRESPred website (http://196.1.114.46:1800/IRESPred/Typer/IRESPred.html).

**Fig. 5:**
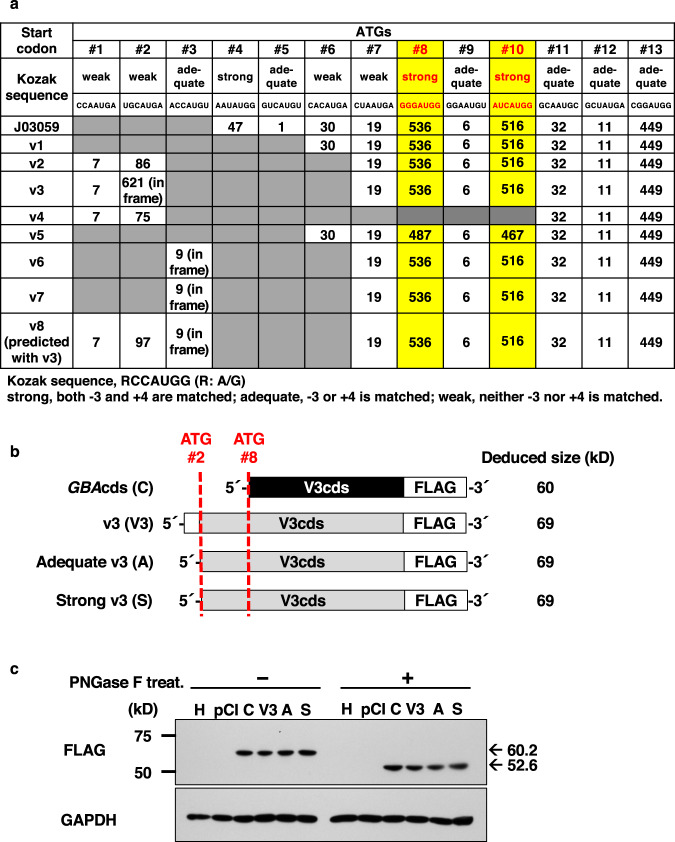
Upstream ATG, uORF, and Kozak sequences. **a** Positions of uATGs, uORFs, and their Kozak sequences within the region from exon 1 to exon 5. The ATGs are numbered from the 5’ ends of the P1 promoter-controlled transcripts. Two reported start sites (#8 and #10) are indicated in red, and the columns are highlighted in yellow^9,20^. The numbers in the table are the predicted sizes of the ORFs for the indicated ATGs on the top line. **b** Schematic of three kinds of v3 constructs inserted into expression vectors. FLAG tags were added to all constructs at the 3’ end. Top line, CDS only; second line (C), CDS with the original 5’ UTR containing a weak Kozak sequence (V3); third line, CDS with a partial 5’ UTR containing an adequate Kozak sequence (A); fourth line, CDS with a partial 5’ UTR containing a strong Kozak sequence (S). **c** FLAG-tagged GCase expression with or without glycosylation in HEK293 cells. GCase protein expression was detected by western blot analysis with anti-FLAG antibodies. C, V3, A, and S indicate the same as in **b**. pCI, empty vector; H, HEK293 cells without transfection. The molecular sizes are indicated on the right side. The experiment was performed in triplicate and the representative data was shown.

Studies have reported that translation was initiated through either cap-dependent or cap-independent mechanisms^20–22^. Cap-dependent translation was stimulated and scanned through an association of the cap-binding protein complex with ribosomal subunits^23^. Moreover, the *GBA* gene sequence analysis revealed that the 5’ UTR driven by the P1 promoter was uniquely long and contained 13 potential translation start codons (AUGs). Therefore, to confirm the possible cap-dependent translation, first, we analyzed the surrounding sequence motifs. As shown in Fig. 5a, their open reading frame (ORF) sizes were deduced to range from 1 amino acid to 621 amino acids. Furthemore, the sequence near the start codon AUG was called the Kozak consensus sequence (with −3 as A or G and +4 as G)^24–26^. Based on the sequence characteristics, start codons of ATG4, ATG8, and ATG10 had strong Kozak sequences (Fig. 5a). Of these sequences, ATG4 was the first ATG appearing in exon 1 of the reference sequence J03059^7^. However, it initiated an upstream ORF composed of 47 amino acids that terminated with a stop codon before ATG8. In contrast, ATG8 and ATG10 corresponded to the two known translation start codons, which were also demonstrated to mediate efficient translation in vitro^27^. Therefore, we conclude that P2 promoter-driven transcripts were translated via the cap-dependent machinery.

In the 5’ UTR of P1 promoter-driven transcripts, six ATG codons (ATG1, ATG2, ATG3, ATG7, ATG11, and ATG12) with weak or adequate Kozak sequences have been identified, appearing with a stop codon shortly after initiation (Fig. 5a). With variant v3, we observed that although the triplet codon from ATG2 was connected to ATG8 and ATG10 in-frame, it potentially initiated an extended ORF encoding 621 amino acids (Fig. 5a, b). Therefore, to confirm whether this ORF can be translated, three expression vectors were constructed with original (weak), adequate or strong Kozak sequences in front of ATG2 at the 5’ UTR of the v3 *GBA* sequence. To detect the translated products, a FLAG tag was added at the C terminus of the GCase coding sequence (CDS) (Fig. 5b). The translation products’ deduced sizes for the sequences beginning at ATG2 and ATG8 were 69 kD and 60 kD, respectively. Subsequently, we transiently transfected these constructs into HEK293 cells, after which western blot analysis was performed with or without Peptide -*N*-Glycosidase F (PNGaseF) treatment. Figure 5c demonstrated that the translation products of v3 either with or without glycosylation was only one band, beginning from ATG8. Hence, P1 promoter-driven *GBA* transcripts can be initiated through cap-independent translational machinery.

### Cap-independent translational control mediated by cell-type-specific internal ribosome entry site (IRES) activities

**Fig. 6:**
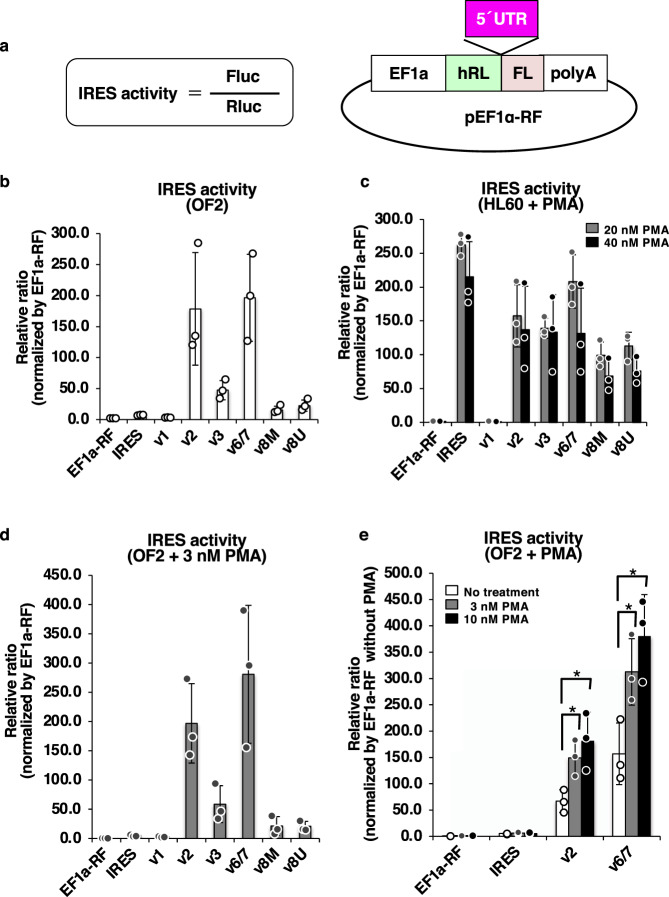
IRES activity in the 5’ UTRs of *GBA* variants. **a** Formula for estimation of IRES activity by bicistronic reporter assay and the reporter construct. The 5’ UTRs of *GBA* variants (pink box) were inserted into the pEF1a-RF vector. EF1a, EF1α promoter; hRL, *Renilla* luciferase CDS; FL, *firefly* luciferase CDS. **b** IRES activity in OFs. **c** IRES activity in HL60-derived macrophages (HL60 + PMA). Gray bar, 20 nM PMA; black bar, 40 nM PMA. **d** IRES activity in OFs treated with 3 nM PMA. **e** Effects of PMA on IRES activity in OFs. White bar, no treatment; gray bar, 3 nM PMA; black bar, 10 nM PMA. Each experiment was performed in triplicate. *p < 0.05. Data represent the mean ± STDEV.

Recently, cellular IRESs have received attention as unique translational machinery elements that cap-independently function^22,28^. For example, the IRES site at the 5’ UTR of the *Hox* gene has been required to translate the Hox protein during body patterning^29^. Therefore, to examine whether the IRES system was involved in the translation of P1 promoter-driven *GBA* transcripts, we established a reporter system of IRES activity. First, we used a bicistronic vector with a dual luciferase assay system (Fig. 6a and Supplementary Fig. 3a). Then, for vector construction, the strong EF1a promoter was used to initiate cap-dependent translation, after which its activities were monitored by assessing Renilla luciferase activities. Finally, IRES-dependent translation activities were measured by evaluating firefly luciferase activities.

First, we examined the IRES activity of *GBA* variants in OF2 cells (Fig. 6b). Interestingly, although the activity of the positive control encephalomyocarditis virus (EMCV) IRES region (EMCV-IRES) construct was approximately 6-fold higher than that of the negative control, 5’ UTRs of variants v2, v3, v6/7, and v8 (v8M and v8U) showed much higher IRES activity levels than the positive control in OF2 cells. Remarkably, variants v2 and v6/7 exhibited extremely high IRES activities, approximately 200-fold higher than the negative control. However, variant v1 had little IRES activities (only 1/3 of IRES activity in the positive control).

Next, we analyzed the IRES activity of *GBA* variants in iMacs (Fig. 6c). Strikingly, the activity of EMCV-IRES, a positive control, was much higher in iMacs than in OF2 cells and was virtually 250-fold higher than that of the negative control. Furthermore, robust IRES activities were observed for all P1 promoter-driven variants in iMacs (variants v2 and 6/7 and variants v3, v8M, and v8U). However, the P2 promoter-driven variant v1 had little IRES activity in iMacs. As shown, these IRES activity profiles were reproduced in iMacs treated with different concentrations of PMA (20 nM and 40 nM, Fig. 6c).

Subsequently, to examine the effect of PMA treatment on IRES activity, we also treated OF2 cells with PMA. Then, the suitable concentration of PMA treatments for OF2 cells was determined by assessing the expression of known PMA target genes (*Cox2* and *Mmp1*)^30,31^ by RT-PCR (Supplementary Fig. 3b). The patterns of IRES activity among the variants in PMA-treated OF2 cells were not different from those in untreated OF2 cells (Fig. 6b, d). However, although the positive control did not show any enhancement of IRES activity, the IRES activities of v2 and v6/7 in OF2 cells were more than 2-fold enhanced through PMA in a dose-dependent manner (Fig. 6e). Furthermore, GCase protein levels in OF2 cells did not differ by PMA treatments (Supplementary Fig. 3c). Therefore, we observed IRES activity in the 5’ UTRs of P1 promoter-driven transcripts, showing their function as novel translational machinery of *GBA* expression.

### Analysis of cap dependency through rapamycin treatments

**Fig. 7:**
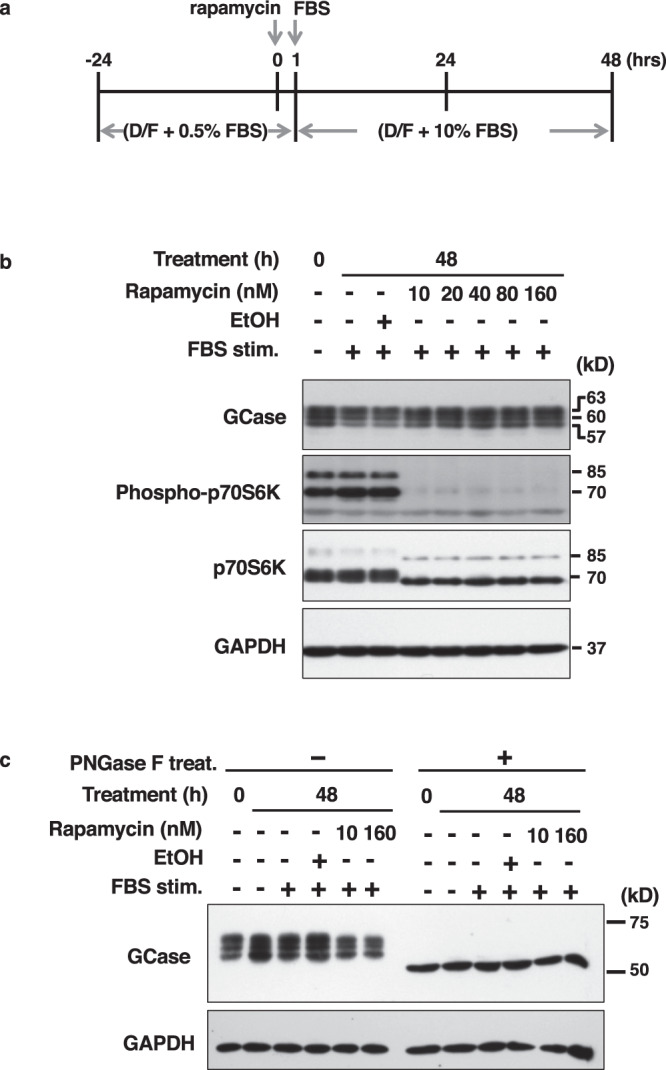
GCase translation in OF2 cells after rapamycin treatment. **a** Diagram of sample preparation. OF2 cells were precultured in media with 0.5% FBS for 24 h before rapamycin treatment. The cells were treated rapamycin for 1 h, FBS was added up to 10% and maintained for 48 h. **b** Results of GCase expression in rapamycin-treated OF2 cells, as determined by western blot analysis. EtOH; 0.1% ethanol, FBS stim.; FBS stimulation. **c** Confirmation of the GCase expression remove its glycosylation. Each experiment was performed in the triplicate and representative data was shown.

As we shown in Fig. 4a, although both OFs and DFs expressed P1 promoter-driven transcripts, P2 promoter-driven transcripts were also detected. Therefore, we examined how much cap-dependent translation translated *GBA* gene transcripts in OFs. Since rapamycin is a well-known inhibitor of cap-dependent translation^32^, we also examined GCase protein expression in OF2 cells treated with or without rapamycin for 48 h (Fig. 7a). As shown, we detected three bands with GCase antibodies by western blot analysis, sizes of which were 63, 60, and 57 kD (Fig. 7b). Furthermore, to confirm whether the three bands were different isoforms or glycoforms, we conducted PNGase F treatment to remove glycosylation. However, only one band was detected in all conditions of this experiment (Fig. 7c). Notably, the intensity of GCase was maintained regardless of rapamycin treatments (no gross changes were observed). Besides, the phosphorylated form of the ribosomal protein p70S6 kinase (phosphor-p70S6K) was practically undetectable after rapamycin treatments as a control (Fig. 7b). Therefore, these results demonstrated that GCase protein synthesis was not affected by rapamycin treatments in OFs and that cap-independent translation can maintain GCase expression levels.

Additionally, it was observed that although rapamycin may inhibit the phosphorylation of TFEB to allow nuclear translocation and transcription of target genes, including the *GBA* gene^33^, the lack of an increase in GCase protein level exists. One possible cause is that rapamycin’s induction of TFEB activation was proposed to regulate P2 promoter activation, and lower in OFs which were used for these experiments. Nevertheless, further investigations should be done to confirm this issue.

### Characterization of the 3’ UTR

**Fig. 8:**
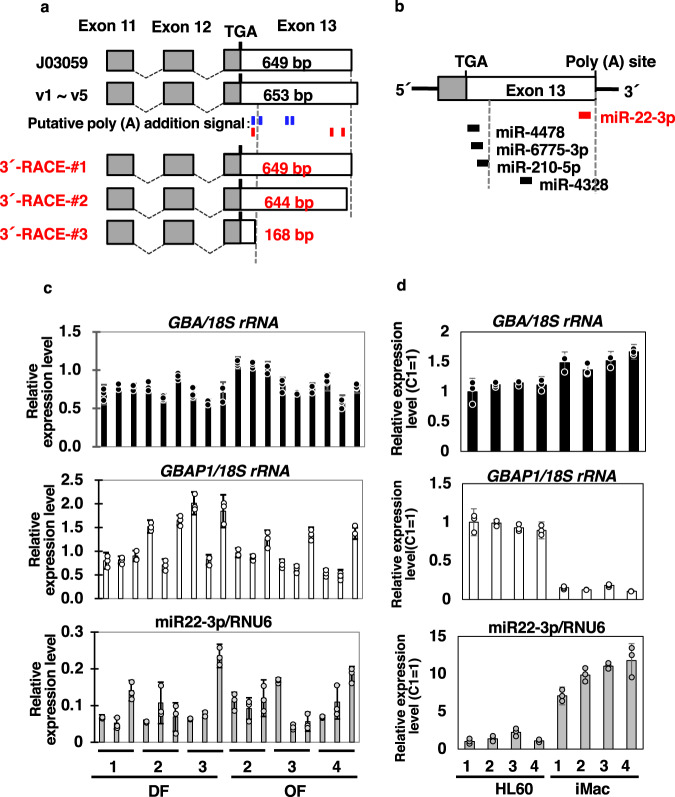
Analyses of the *GBA* 3’ end. **a** Summary of *GBA* 3’-RACE. The top 2 lines indicate the known structures of J03059 and GBA variants v1 to v5. #1–3 are the results of this study. The boxes indicate the exons. Gray, coding region; white, UTR. The number is the size of exon 13. In the middle, the positions of putative poly (A) addition signals are indicated in blue (Horowitz, et al.^8^) and red (Beaudoing et al.^32^). **b** In silico analysis of miRNA binding to *GBA*. Red, miR22-3p based on Straniero et al.^10^; black, database search results obtained using miRDB. **c**, **d** Gene expression of *GBA*, *GBAP1* and miR22-3p in fibroblasts (**c**) and HL60 derivatives (**d**). The qPCR results for *GBA* and *GBAP1* were normalized to *18* *S rRNA* expression, whereas miR22-3p was normalized to *RNU6* expression, respectively. In **d**, after normalized expression value, calibrated as a relative quantity to no-treated HL60 (C1). Black bar, *GBA*; white bar, *GBAP1*; gray bar, miR22-3p. Each experiment was performed in triplicate. Data represent the mean ± STDEV.

The 3’ UTR structures of mRNAs have been reported to regulate mRNA’s stability, localization, and translation^34^. Therefore, we analyzed the 3’ ends of *GBA* mRNA using 3’ RACE analysis, and three different 3’ UTR lengths were detected (Fig. 8a). Furthermore, although a previous report indicated three potential polyadenylation signal sites^8^, we identified another one. Subsequently, we predicted two additional polyadenylation signal sites in the 3’ UTR of exon 13 using different criteria^35^. Two of these six polyadenylation signal sites matched our 3’ RACE results.

Recently, the function of pseudogenes has been focused. Pseudogene-derived transcripts were trapped and degraded upon miRNA binding, resulting in protection from corresponding authentic gene degradation, called ceRNA or RNA sponge effects^11^. Besides, to downregulate *GBA* and *GBAP* mRNA levels, miR22-3p has been reported as a functional miRNA that binds to their 3’ UTR^10^. Therefore, we confirmed the miR22-3p binding site by analyzing our 3’ UTR sequence using miRBase (http://www.mirbase.org/). Similarly, we identified four potential miRNA binding sites. However, the miR22-3p binding site was not found (Fig. 8b). Hence, we evaluated whether the expression level of *GBA*, *GBAP1*, and miR22-3p can be observed in the ceRNA network, through qPCR analysis using reported primers^10^. Notably, the reported primers recognized the common sequence among *GBA and GBAP1* variants, respectively^10^. So, we compared the total levels of *GBA transcripts* and that of *GBAP1*. As shown in Fig. 8c, d, miR22-3p expression was not correlated with the total expression of either *GBA* or *GBAP1* in fibroblasts of our system. Interestingly, we also observed that the expression level of total *GBA* and miR22-3p versus *GBAP1* transcripts formed a reciprocal pattern between HL60 and iMacs. Thus, it was suggested that another function of *GBAP1* exists in iMacs.

## Discussion

Mechanisms of functional GCase expression are crucial for the catabolism of glucocerebroside in lysosomes. However, the molecular mechanisms of cell-type-specific gene regulation during GCase expression have remained unclear^8,19^. Based on our findings, we proposed a redefined *GBA* gene structure. Additionally, we provided new evidence for cell-type-specific regulatory mechanisms of *GBA* gene expression at the levels of transcription and translation levels.

### Cell-type-specific promoter regulation

It has been reported that although the *GBA* gene is constitutively expressed in all body cells, it is differentially expressed^36,37^. Our data demonstrated that expression of the *GBA* gene can be controlled by differential promoter usage. The P1 promoter sequence of the *GBA* gene is TATA-less and contains many Sp1 binding sites, commonly observed in housekeeping gene sequences^38^. Therefore, this study demonstrated that P1 promoter-driven transcripts were present in fibroblasts (Fig. 4a). Alternatively, mRNA levels transcribed from P2 promoter of the *GBA* gene were more than 5-fold higher in HL60 cells with PMA-mediated macrophage differentiation than in untreated HL60 control cells (Fig. 4b). We also observed that the P2 promoter region contains TPA response elements and tandem TFEB binding sites near the putative TATA box (Fig. 2b, red boxes and green underlines). TFEB is a positive transcriptional regulator of the *GBA* gene^18^, and TFEB can be activated through the PMA-PKC pathway^39^. Hence, since PKCβ has been reported to be a key regulator of PMA-induced macrophage differentiation of HL60 cells^40^, TFEB activation is proposed to be involved in PMA-triggered macrophage induction. Supporting this possibility, a previous study has demonstrated that TFEB can regulate macrophage activity^41,42^. We also observed an approximately 1.6-fold enhanced *TFEB* mRNA expression levels in iMacs compared to the original HL60 cells (Supplementary Fig. 2c). However, further experiments are required to elucidate whether TFEB and PMA are coregulators of the cell type-specific P2 promoter.

Additionally, two alternative promoters have been reported previously^9^, P2 and P1 (rom the 5’ end). Our redefined structure placed these promoters in the opposite order. They also showed that both promoter-derived *GBA* transcripts were ubiquitously expressed in various tissues and thus behaved like housekeeping genes transcripts^9^. However, we obtained different results in fibroblasts and iMacs (Fig. 4). These contradictory findings are considered to be due to experimental conditions at the tissue or cell-type level. Moreover, since tissues are composed of heterogeneous cell types, gene expression can be partly offset.

### Cell-type-specific translational regulation

Translation initiation is regulated by two basic types of mechanisms: cap-dependent and cap-independent^20–22^. For the former, a scanning model and a scanning-free model have been proposed. The latter involves IRESs, cap-independent translational enhancer, and RNA modification. Each mechanism is controlled by structural-dependent regulators^20–22,43^. Similarly, in this study, we observed that two processes, mainly cell-type-specifically, influenced the translation of *GBA*. First, constitutive P1- and P2 promoter-driven transcripts were translated in fibroblasts by cap-independent IRES and IRES-independent mechanisms (Figs. 4a and 7b, c). Second, inducible P2 promoter-driven transcripts were translated by IRES-independent processes in macrophages (Figs. 4b and 6c).

The IRES structure in gene transcripts is one of the well-studied RNA secondary structures involved in translation, and 10–15% of mammalian mRNAs are predicted to contain IRESs^20,44^. Therefore, cellular genes with IRESs can have two major physiological functions^45^. One is to keep less translation of the highly structured 5’ UTR when a cap-dependent translation is fully active under physiological conditions. The other is, when cap-dependent translation is inhibited under pathophysiological and stress conditions, translation switches from cap-dependent to cap-independent forms through the regulation of IRES *trans-*acting factors (ITAFs)^45,46^. Moreover, most cases of cellular IRES regulation have been reported to correspond to the latter status^45,46^. Remarkably, in this study, we observed both scenarios with *GBA* expression.

Based on the first IRES-mediated case, we identified novel and functional IRES activities in 5’ UTRs of P1 promoter-driven variants, indicating that the cap-independent translational regulation of GCase was constitutively active in fibroblasts (Fig. 6b, d, e).

It has also been reported that although the median length of 5’ UTRs in known human mRNAs was 218 nucleotides^20^, P1 promoter-driven variants, especially v2–v4 and v6–v8, had notably longer 5’ UTRs (331 to 547 nucleotides) (Table 1). Besides, long ribosome scanning at 5’ UTRs is influenced by GC content and free energy (Δ*G*)^20^. Nevertheless, high GC levels and the highly negative folding Δ*G*s of 5’ UTRs often cause 5’ UTR RNA secondary structures to form, resulting in inhibition of scanning^20^. As shown in Table 1, the 5’ UTR sequences of P1 promoter-driven *GBA* transcripts had Δ*G* values of over −100 kcal/mol, which can be disrupted scanning and progressing ribosomes^47,48^. Moreover, no translated product of the longest ORF of variant v3 was detected, even when the initially weak Kozak sequence was replaced with a strong sequence (Fig. 5c). Therefore, results suggested that the unusually long 5’ UTRs of *GBA* gene variants formed secondary structures, preventing the execution of ribosomal scanning.

Regarding the second IRES-mediated case, we observed *GBA* regulation in iMacs. Based on our findings shown in Fig. 4b, the major endogenous transcripts were P2 promoter-derived variants whose translation was proposed as cap-dependent due to sequence properties. However, we also observed cellular IRES activity in the 5’ UTRs of P1 promoter-driven *GBA* transcripts in iMacs transfected with an exogenous bicistronic vector (Fig. 6c). Furthermore, we observed the activation of IRES elements in the 5’ UTRs of P1 promoter-driven *GBA* transcripts, showing activity levels comparable to those of the EMCV-IRES element (the positive control) in iMacs (Fig. 6c). These results suggested that ITAF activity was inducible during macrophage differentiation mediated by PMA stimulation. Interestingly, although PMA stimulation also enhanced the IRES activity of variant-specific 5’ UTRs of v2 and v6/7, those of EMCV-IRES or other P2-driven 5’ UTRs in OF2 cells was not improved (Fig. 6d, e). Hence, iMacs are proposed to contain the same or similarly positive ITAFs as EMCV-IRES, e.g., polypyrimidine tract-binding protein (PTB)^49^, or iMac-specific cellular ITAFs, such as p53, APAF1, and BiP^46^. Alternatively, in fibroblasts, an extremely high level of IRES activity was observed compared to that of the control EMCV-IRES element (Fig. 6b). Therefore, these findings propose that fibroblast-specific ITAFs differed from EMCV-IRES and iMac ITAFs and played different roles in sphingolipid metabolism. However, further analysis is required to understand IRES regulation by ITAFs better. A previous study has notably reported TCP80/NF90 as a negative regulator of *GBA* translation, where Xu et al. showed that TCP80/NF90 interacts with the *GBA* mRNA coding region^50^, and confirmed alters mRNA binding to polysomes to inhibit translation through using an expression vector containing the 5’ UTR and CDS of *GBA* in Sf9 cells^51^. TCP80 has also been reported as a positive ITAF of the p53 response to DNA damage in cancer cells^52^. Therefore, TCP80 is proposed to be one of the GBA ITAFs. Collectively, these data indicate that *GBA* expression was cell-type-specific and finely regulated constitutively/inducibly through IRES activities.

### Cell-type-specific GBAP1 functions

A previous report has also demonstrated that the promoter activity of *GBAP1* was negligible through a CAT assay in the epithelial cell lines (HeLa or hepatoma cells) and a B cell line, prepared using a 650-bp *Sac*I fragment, containing the original exon one as the promoter region^7^. However, as shown in Fig. 8c, we detected *GBAP1* expression in OFs. This discrepancy can be due to the three possibilities. The first possibility is the cell-type specific expression of *GBAP1*. The second is the sensitivity of the detection system, either the CAT assay or qPCR. Specifically, qPCR can amplify the transcripts and quantify the original transcripts’ levels. The third is promoter usage. It has been demonstrated that the expression level of *GBA* and *GBAP1* is cell-type dependent among several cell lines, including HeLa, HepG2, and GM06895^10^. As observed, although the expression level of *GBAP1* was extremely low in HeLa, HepG2 and GM06895, it remained detectable through qPCR. They also demonstrated *GBAP1* structure and splicing variants^10^, showing that an additional promoter region can be also exist for *GBAP1*. Therefore, the differential promoter usage can cause discrepancies.

A pseudogene was an initially defective and non-functional gene. However, their functions were recently focused on and discussed as natural antisense transcripts (involved in epigenetic regulation, RNA editing, processing, etc.). They were also known as “competitive endogenous RNA (ceRNA),” “microRNA sponge,” and competing “stabilizing factors,” and forming chimeric transcripts^53,54^. Furthermore, possible *GBAP1* functions have been reported as ceRNA to keep the *GBA* expression high. This ceRNA function has also been described in OCT4^55^, and other genes^53,54^. Nevertheless, our finding demonstrated the reciprocal expression of both *GBA* and miRNA22-3p versus *GBAP1* between HL60 cells and iMac. The reduction of *GBAP1* level proposed to be the result of sponging, or another function of *GBAP1* can be working on iMac, through HL60-induced macrophage differentiation. Yet these possibilities need to be clarified in the future.

### Biological questions and future perspectives

This study revealed that *GBA* expression was cell-type-specifically regulated by combining *cis*- and *trans*-regulatory mechanisms at the transcriptional and translational levels. Our findings suggested new regulatory aspects of *GBA* expression and provided essential information to enhance our understanding of the cell-type-specific role of GCase in glycosphingolipid metabolism. Therefore, this study is proposed to support the development of new diagnostic tools or therapeutics for patients with GD in the future.

## Methods

### Cell culture

The human DFs (DF1, DF2, and DF3 cells) and human OFs (OF2, OF3, and OF4 cells) used in this study have been previously described^13,56^. In brief, the human DFs were obtained from the Health Science Research Resources Bank (TIG-110, TIG-111, TIG-114, Osaka, Japan), and cultured in Eagle’s MEM supplemented with 10% FBS^13,56^. Human OFs were isolated from the buccal mucosal tissues obtained from healthy volunteers, and cultured in Dulbecco’s Modified Eagle Medium with 10% FBS^13,56^. Research approval was obtained from the Institutional Research Ethics Committee of Tokushima University (Project No. 708) based on the individual informed consent and written agreement. The human promyelocytic leukemia cells (HL60 cells)^57^ used in this study have also been described previously^14–16,58^. In brief, HL60 cells were provided from RIKEN Resource Center through the National Bio-Resource Project of the Ministry of Education, Culture, Sports, Science and Technology, Japan^57^, and maintained in a RPMI-1640 medium with 10% FBS^14–16,58^.

### Determination of the 5’ ends of *GBA* by RLM-RACE and cDNA sequencing

Total RNA was isolated from DF2 cells using TRI Reagent (Molecular Research Center, Cincinnati, OH, USA), and 2.5 μg of RNA was used for the determination of 5’ ends of *GBA* with a GeneRacer^TM^ Kit (Invitrogen, Carlsbad, CA, USA) following the manufacturer’s protocol. The GeneRacer^TM^ Kit protocol is based on RLM-RACE methods, which can selectively ligate RNA oligonucleotides to the 5’ ends of de-capped mRNA using T4 RNA ligase^59–61^. cDNAs of *GBA* were synthesized after reverse transcription (RT) using oligo dT primers. Then, the 1st round of PCR was performed with the GeneRacer^TM^ 5’ primer, 5’-CGACTGGAGCACGAGGACACTGA-3’, and a reverse *GBA-*specific primer, 5’-GTATCTTCCTCTGGGAGGCTGAAG-3’, under the following conditions: 1 cycle of denaturing at 98 °C for 10 s, 20 cycles of denaturing at 98 °C for 10 s, annealing at 58 °C for 5 s, and extension at 72 °C for 1.5 min; and 1 cycle of extension at 72 °C for 5 min. PrimeSTAR^®^ HS DNA Polymerase (Takara Bio, Shiga, Japan) was used as a thermostable DNA polymerase. Next, 2 μl of each 1st-round PCR product was used as a template, and a 2nd round of PCR was performed with the GeneRacer^TM^ 5′ nested primer, 5′-GGACACTGACATGGACTGAAGGAGTA-3′, and a *GBA-*specific 5′ nested primer, 5′-TGGGTACCCGGATGATGTTATATCCG-3′ under the following conditions: 1 cycle of denaturing at 98 °C for 10 s; 25 cycles of denaturing at 98 °C for 10 s, annealing at 58 °C for 5 s, and extension at 72 °C for 1.5 min; and 1 cycle of extension at 72 °C for 5 min. The 2nd-round PCR products were extracted using a Wizard^®^ SV Gel and PCR Clean-Up System (Promega, Madison, WI, USA), ligated into the pGEM-T^®^ Easy vector (Promega), and transformed into *Escherichia coli* JM109. The 5′ ends of *GBA-*containing vectors were amplified and purified using a QIAprep Spin Miniprep Kit (QIAGEN, Hilden, Germany). The DNA fragments of the 5′ ends of *GBA* were labeled using a BigDye Terminator v3.1 Cycle Sequencing Kit (Applied Biosystems, Foster, CA) and analyzed with an ABI 3130 Genetic Analyzer (Applied Biosystems). Multiple alignment analysis was performed using GENETYX SV/RC software version 17.0.1 (GENETYX, Tokyo, Japan).

### Expression vectors for *GBA* variants v4 and v5

Coding sequence DNAs of both *GBA* variants v4 and v5 were synthesized with FLAG tag sequence at 3′ end, and individually inserted into pCIneo mammalian expression vector by Vector Builder (Santa Clara, CA, USA). The vector ID is VB200706-1663hkb and VB200630-1074hcz, which can be used to retrieve detailed information about the vector on vectorbuilder.com.

### Neutrophil and macrophage differentiation from HL60 cells

The protocol used for differentiation of macrophages and neutrophils from HL60 cells has been described previously^14–16,58^. In brief, macrophage differentiation was induced by 20 nM phorbol myristate acetate (PMA) treatment for HL-60 (1 × 10^5^ cells/mL) for 24 h. Neutrophil differentiation was induced by 10 µM all-trans retinoic acid (ATRA) for HL-60 (2.5 × 10^5^ cells/mL) for 4 days^58^.

In this study, we named the macrophages induced from HL60 cells “iMacs” and the neutrophils induced from HL60 cells “iNeuts”.

### Determination of gene expression by qPCR

Total RNA was purified from DFs, OFs, HL60 cells, iMacs, and iNeuts, respectively. To detect *GBA* variant expression, the cDNAs were first synthesized in the same way as for RLM-RACE, and then first-round PCR was performed using PrimeSTAR^®^ Max DNA Polymerase (Takara Bio) with the GeneRacer^TM^ 5′ nested primer and GBA ex8-9R to avoid *GBAP* amplification. The PCR conditions were 20 cycles of denaturing at 98 °C for 10 sec, annealing at 55 °C for 5 s, and extension at 72 °C for 10 s. After the PCR products were purified with a Wizard^®^ SV Gel and PCR Clean-Up System (Promega), qPCR was performed using Thunderbird SYBR qPCR Mix (Toyobo Co. Ltd., Osaka, Japan) in a 7300 Real-Time PCR System (Applied Biosystems). The gene specific primers for RLM-RACE and new exon confirmation are shown in Supplementary Table 1. The expression levels of the variants were analyzed by the ΔΔCT method. Each target transcript was normalized to those of either *glyceraldehyde-3-phosphate dehydrogenase* (*GAPDH*; for DFs and OFs) or *peptidyl prolyl isomerase A* (*PPIA*; for HL60 cells and their differentiated cells), and then, calibrated to DF2 or control HL60 cells (no treatment), respectively. All data were obtained in triplicate. The positions, sequences, and combinations of the *GBA* variant-specific primers are shown in Supplementary Fig. 2a and Supplementary Tables 2 and 3, respectively.

### Analysis of GCase protein expression

First, we prepared a human *GBA* expression plasmid by inserting the *GBA* CDS into a mammalian expression vector, pCI-neo (Promega), and transfected HEK293 cells as the positive controls. We then loaded twenty micrograms of total protein from cultured cells, such as HL60 cells, iMacs, and iNeuts, in SDS lysis buffer and 7.88 μg of total protein from positive control cells in SDS lysis buffer onto 8% or 10% SDS-polyacrylamide gels and performed SDS-PAGE analysis. After all proteins were transferred to a PVDF membrane, the membrane was soaked in blocking solution (5% skim milk in TBST) at 4 °C overnight. Two anti-glucocerebrosidase primary antibodies (G4171, Sigma–Aldrich; 2E2, Abnova) were used at 1:10,000 or 1:1000 dilutions, and a horseradish peroxidase (HRP)-linked anti-rabbit IgG secondary antibody (GE Healthcare, Chicago, IL, USA) was used at a 1:5,000 dilution. The signals were detected with Immobilon Western Chemiluminescent HRP Substrate™ (Millipore, Billerica, MA, USA) or Clarity Max^TM^ Western ECL substrate (Bio-Rad, Hercules, CA, USA) and exposed to Fuji medical X-ray film (Fujifilm, Tokyo, Japan). For normalization, the membrane was reprobed for β-Actin detection using a monoclonal anti-β-Actin primary antibody (clone AC-15, Sigma-Aldrich) at a 1:10,000 dilution and an HRP-linked anti-mouse IgG secondary antibody (GE Healthcare) at a 1:5,000 dilution. The expression levels were analyzed using a Bio-Rad ChemiDoc XRS System™ (Bio-Rad). When rapamycin effects on GCase protein expression in OF2 were performed, we detected p70S6k protein with or without phosphorylation and GAPDH expression using anti phospho-p70S6k (Thr389) antibody (9205, Cell Signaling Technology), anti p70S6k antibody (2708, Cell Signaling Technology), and anti GAPDH (14C10) antibody (2118, Cell Signaling Technology), respectively.

### PNGase F treatment

Cell lysates were prepared in cOmplete Lysis-M reagent with protease inhibitor cocktail (Roche, Basel, Switzerland) as following the manufacture’s protocol. Then the lysates were treated with PNGase F (New England Biolabs Ipswich, MA, USA), according to the manufacture’s instruction. In brief, 20 μg of total protein were denatured at 100 °C for 10 min in denaturing buffer, then added PNGase F, 10% NP-40, and Glyco buffer 2, and incubate at 37 °C for 1 h. The samples were applied for western blot analysis as described above.

### Analysis of GCase activity

HL60 cells, iMacs, and iNeuts were harvested in citrate-phosphate buffer (pH 5.4) containing 0.25% Triton X-100 (Wako Pure Chemical, Osaka, Japan) and disrupted by sonication with a Sonifier Model 250 (Branson Ultrasonics Corporation, Danbury, CT, USA). After centrifugation (10,000 × *g* for 30 min), the supernatants were collected as the extracted samples for analysis of GCase activity. The protein concentration in each cell extract was determined using a Pierce BCA Protein Assay Kit (Pierce Biotechnology, Rockford, IL, USA).

To analyze GCase activity, we used the methods in the report of Mazzulli et al.^62^, with some modifications. In brief, each cell extract was incubated for 40 min at 37 °C in 100 μl of assay buffer (0.25% Triton X-100, 2.5 μg/μl taurocholic acid, 1 mM ethylenediaminetetraacetic acid (EDTA), 1% bovine serum albumin (BSA), 1 mM 4-methylumbelliferyl β-D-glucopyranoside (4-MU-glc), citrate-phosphate buffer, pH 5.4), and the reactions were stopped by the addition of 100 μl of 1 M glycine-NaOH (pH 12.5). Fluorescence (Ex = 355 nm, Em = 460 nm) was measured with a Varioskan Flash spectral scanning multimode reader (Thermo Fisher Scientific, Waltham, MA, USA). The chemicals were obtained as follows: Triton X-100, NaOH, and phosphate were obtained from Wako Pure Chemical; BSA, taurocholic acid, and 4-MU-glc were obtained from Sigma–Aldrich; and citrate, glycine, and EDTA were obtained from Nacalai Tesque (Kyoto, Japan).

### Expression vector constructs of *GBA* v3 with various Kozak sequences

To confirm whether the *in-frame* uATG of *GBA v3* was able to start translation, we inserted strong, adequate, and weak Kozak sequences in front of the v3 CDSs in constructs by 3 step-cloning. First, forward primers with the structure *Sac*II–*Nhe*I–(Kozak sequence)–(following 17 bases) and a reverse primer that selected the sequence upstream of the *Hind*III site in *GBA* were used for PCR cloning with PrimeSTAR^®^ GXL DNA Polymerase (Takara Bio), and the products were then cloned into the pGEM-T Easy vector (Promega). In parallel, we cloned the coding region of *GBA* with a Flag tag added to the 3’ end into the pGEM-T Easy vector. Next, both clones were digested by *Sac*II and *Hin*dIII, and the 5’ end of *GBA* was ligated with three types of Kozak sequences to the *Sac*II- and *Hin*dIII-digested GBA CDS with Flag in the pGEM-T Easy vector. Third, the three types of plasmids (in the series of *GBA v3* constructs in pGEM-T Easy) were redigested by *Nhe*I and *Eco*RI and cloned into the pCIneo vector (Promega). The identity of each construct was confirmed by sequencing. The primer sequences are listed in Supplementary Table 4.

All constructs were individually transfected into HEK293 cells using X-tream GENE HP (Roche), and total cell lysates were separated by SDS-PAGE (8% separating gels). After western blotting, FLAG-tagged proteins were detected using a monoclonal anti-FLAG^®^ antibody (clone M2, Sigma–Aldrich; 1:10,000), similar to the methods for GCase protein analysis.

### IRES activity assay for the 5’ UTRs of GBA variants

A bicistronic vector was prepared through several steps. First, the EF1α promoter was amplified from the pBApo-EF1α Pur vector (Takara) by PCR using a forward primer that added an *Nhe*I site (*Nhe*I-EF1α-F: 5′-CTAGCTAGCATTCGTGAGGCTCCGGTGC-3′) and a reverse primer that selected the sequence upstream of the *Hin*dIII site in the template pBApo-EF1α Pur vector (EF1α-*Hin*dIII-R: 5′-CCCAAGCTTCACGACACCTGAAATGGAAG-3′), and the product was cloned into the pGEM-T Easy vector (Promega). In parallel, EMCV-IRES was amplified from pIRES2-AcGFP1 by PCR using the forward primer *Nco*I-*Xba*I-*Spe*I-IRES-F (5′-CCATGGTCTAGAACTAGTGCCCCTCTCCCTCCCCC-3′) and the reverse primer IRES-*Spe*I-*Nco*I-R (5′-CCATGGACTAGTTTGTGGCCATATTATCATCGTG-3′), and the product was cloned into the pGEM-T Easy vector. Then, the EMCV-IRES fragments were digested by *Nco*I and cloned into the pEF1α-Luc vector. The EF1α promoter was digested by *Nhe*I and *Hin*dIII and cloned into the pGL3-control vector, which contained *firefly* luciferase (producing the pEF1α-FLuc vector). Next, both the phRL-TK vector (Promega) and the pEF1α-FLuc vector were digested by *Hin*dIII and *Xba*I, and the *firefly* luciferase fragment was replaced with the *Renilla* luciferase fragment in the pEF1α-FLuc vector (creating the pEF1α-RLuc vector). Finally, both the pEF1α-RLuc and pEF1α-FLuc vectors were digested by *Xba*I, and the EMCV-IRES-*firefly* luciferase fragment was inserted following the *Renilla* luciferase fragment in the pEF1α-RLuc vector (creating a pEF1α-RLuc-EMCV-IRES-Fluc vector named the pEF1α-RL-IRES-FL vector, positive control). For the negative control, the pRL-IRES-FL vector was digested by *Spe*I, and the IRES fragment (named pEF1α-RF) was removed. The final vector constructs are depicted in Supplementary Fig. 3a.

To analyze the IRES activity of the *GBA* variants, we cloned the 5’ UTRs of *v1, v2, v3, v6/7*, and *v8*, which contained the regions upstream of ATG8, a known translation start site, by PCR using *Spe*I-tagged primers. Then, the 5′ UTR fragments were cloned into the pGEM-T Easy vector, digested by *Spe*I, and recloned into pEF1α-RL-FL. The primer sets are listed in Supplementary Table 5.

Before transfection, 5.2 × 10^4^ OF2 cells/well were seeded in 24-well plates. To prepare PMA-treated OF2 cells, 5.0 × 10^4^ OF2 cells/well were seeded in 24-well plates in DMEM supplemented with 1% FBS. After 24 h, the medium was changed to DMEM supplemented with 1% FBS with 3 or 10 nM PMA, and the cells were cultured for another 24 h. For macrophage induction, 6.0 × 10^5^ HL60 cells/well were seeded in 24-well plates, and 20 nM or 40 nM PMA was added. Each IRES vector (400 ng) was transfected into PMA-induced macrophages differentiated from HL60 cells seeded in 24-well plates with X-tream GENE HD (Roche) (DNA: reagent = 1:2) for OFs, and a Trans-IT-X2 Dynamic Delivery System (Mirus Bio LLC, Madison, WI, USA) (DNA: reagent = 1:5) for macrophages. Twenty-four hours after transfection, the cells were harvested, and IRES activity was analyzed with a Dual-Luciferase^®^ Reporter Assay System (Promega) by Lumat LB 9507 (Berthold technologies GmbH & Co.KG, Württemberg, Germany). IRES activity was calculated as the *firefly* luciferase activity divided by the *Renilla* luciferase activity, and the relative ratios were normalized by the IRES activity of pEF1α-RF. Each assay was performed in triplicate and repeated at least 3 times independently.

### miR22-3p expression

To confirm miR22-3p expression in our system, qPCR was performed using RNA from DFs, OFs, and PMA-induced macrophages in a miScript PCR system (QIAGEN). RT preparation was performed with 500 ng of total RNA and oligo dT primers with universal tags following the manufacturer’s protocol. The quantitative qPCR was performed using THUNDERBIRD^®^ NextSYBR^®^ qPCR Mix (Toyobo) with a CFX Connect Real-Time System (Bio-Rad), and analyzed by the standard curve method. The primer sequences were those reported by Straniero et al.^10^ and are listed in Supplementary Table 6.

### Statistics and reproducibility

For GCase data, significant differences between samples were analyzed with one-way ANOVA followed by Tukey-Kramer multiple comparisons tests. For the other data, significance was evaluated by Student’s *t*-test. *p* < 0.01 was considered to indicate statistical significance. Each data is shown as the mean with an error bar representing the standard deviation, respectively. Showing blots and gels were representative data repeated more than two or three times with independent samples or experiments.

### Reporting summary

Further information on research design is available in the Nature Research Reporting Summary linked to this article.

## Supplementary information

Supplementary Information

Description of Additional Supplementary Files

Supplementary Data 1

Reporting Summary

## Data Availability

The variant accession numbers which we used in this study are as follows: v1, NM_000157; v2, NM_001005741; v3, NM_001005742; v4, NM_001171811; v5, NM_001171812. The partial sequences of human GBA v6, v7, and v8 mRNA that support the findings of this study have been deposited into GenBank through DDBJ under the accession numbers LC050340, LC050341, and LC050342, respectively. Other data supporting the findings of this study are available within the paper and its supplementary information files (included uncropped blots/gels; as Supplementary Fig. 4), and Supplementary Data 1.

## Code availability

The softwares for data analysis in this study were available in the method section. In brief, DNA sequence data were analyzed by GENETYX SV/RC software version 17.0.1 (GENETYX, Tokyo, Japan). ∆G (free energy) was analyzed by the IRESPred website (http://196.1.114.46:1800/IRESPred/Typer/IRESPred.html. The potential miRNA binding sites were found using miRBase (http://www.mirbase.org/). All calculations were done by Microsoft Excel for Mac versin 16.35.
