# Supplementary material for: Redefining GBA gene structure unveils the ability of Cap-independent, IRES-dependent gene regulation
Source: Commun Biol. 2022 Jul 13;5:639. doi: 10.1038/s42003-022-03577-5 (PMC9279297; doi:10.1038/s42003-022-03577-5)
Supplement: Supplementary file 1 — Supplementary Information [file 42003_2022_3577_MOESM1_ESM.pdf]

Communications Biology

# Redefining GBA gene structure unveils the ability of Cap-independent, IRES-dependent gene regulation

Miyoshi K. et al.

## Supplementary Information

Supplementary Figures: 4 (included Uncropped blots/gels)

Supplementary Tables: 6

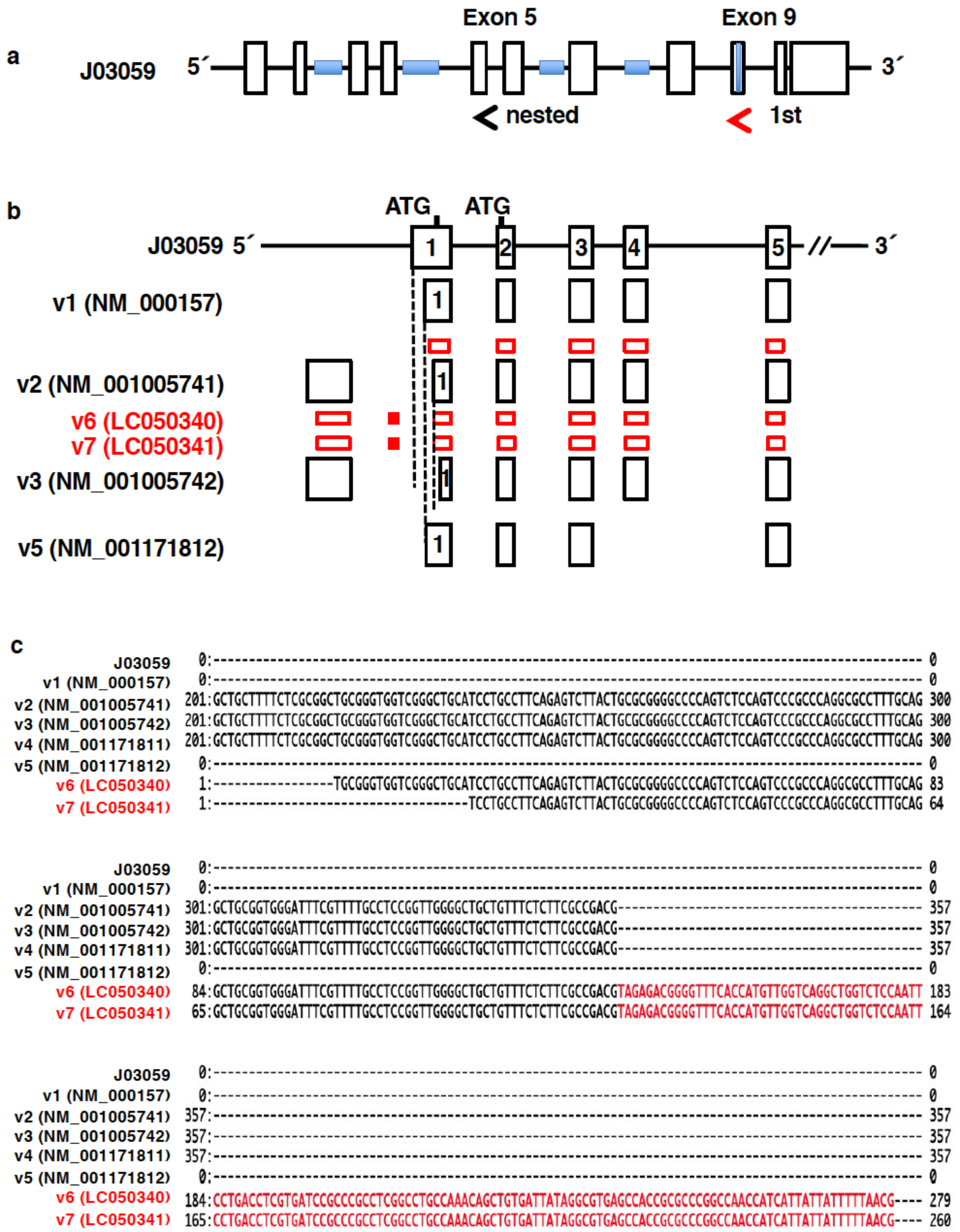


**Supplementary Fig. 1. Determination of the 5′ end of the *GBA* gene in DFs.**

a. The positions of gene-specific 1st-round PCR primers and nested primers for 5′ RACE are shown by the red and black arrowheads, respectively. The blue boxes indicate the *GBA*-specific regions that do not exist in the *GBAP1* gene. b. Summary of the 5′ structures of the *GBA* gene identified by RLM-RACE analysis. The top row shows the exon-intron structure of the reference sequence J03059. The boxes indicate exons, and two translational start sites are shown as “ATG”. The red open boxes are the exons identified by RLM-RACE analysis; the red filled boxes are the novel exons identified in this study. The dashed lines indicate the exon start sites in the different variants. c. Alignment of the 5′ end sequences of the newly identified variants and the five known *GBA* variants to the reference sequence J03059. The sequences of the novel exons are shown in red. The numbers indicate the start and end positions of the variant sequences. (continued to next page)

d

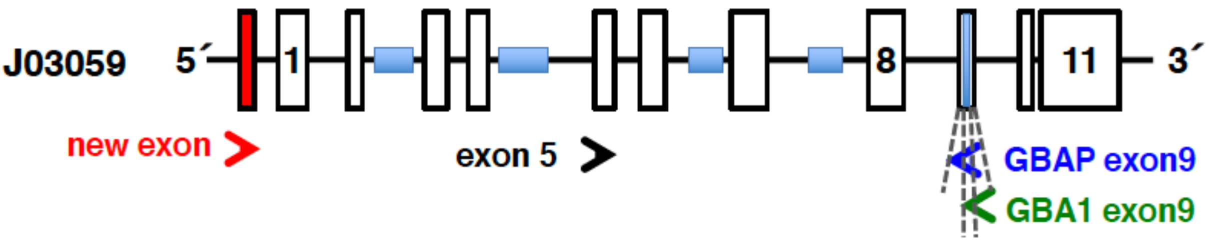


e

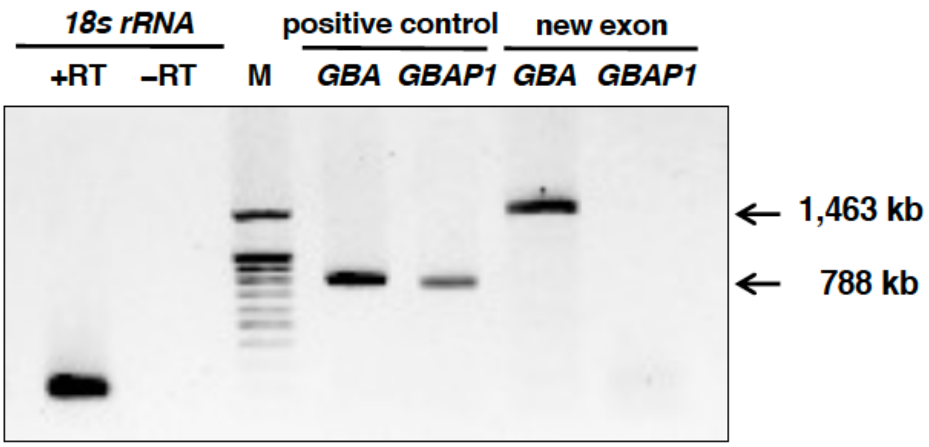


**Supplementary Fig. 1 (continued)**

d. Positions of the primers used for RT-PCR to determine whether the novel exons originated from the *GBA* gene or the *GBAP1* gene. Red box, new exon; blue box, *GBA*-specific region that does not exist in the *GBAP1* gene. The arrowheads indicate the primer positions. The blue arrowhead shows a specific primer for the *GBAP1* gene that does not contain the sequences in the blue box. The green arrowhead indicates a specific primer for the *GBA* gene that is located within the blue box. The primer sequences are listed in Supplementary Table 1. e. Confirmation of the novel exons specific to the *GBA* gene. +RT and –RT indicate that cDNA synthesis was performed with and without reverse transcriptase, respectively. M indicates the DNA size markers. The positive controls amplified exon 5 to exon 9 of *GBA* and *GBAP1*. The new exons were specifically amplified using the new exon forward primer with the exon 9 reverse primer for *GBA* but not *GBAP*1. All PCR products were confirmed by sequencing. (continued to next page)

f

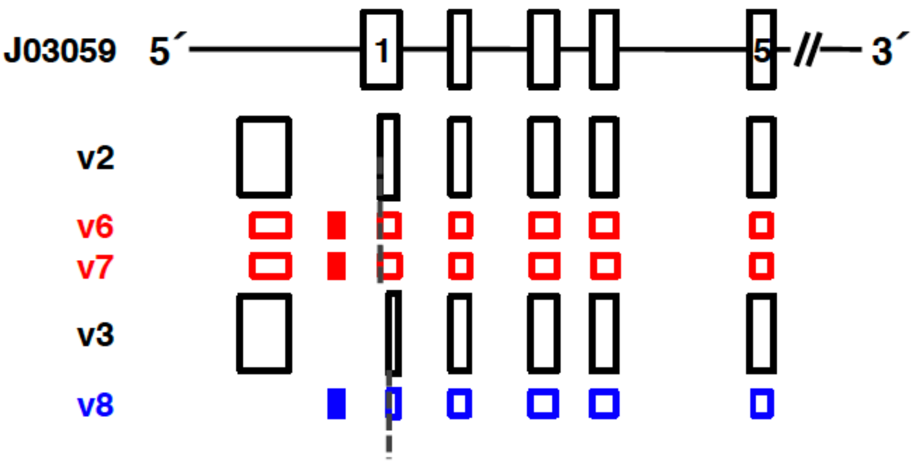


g

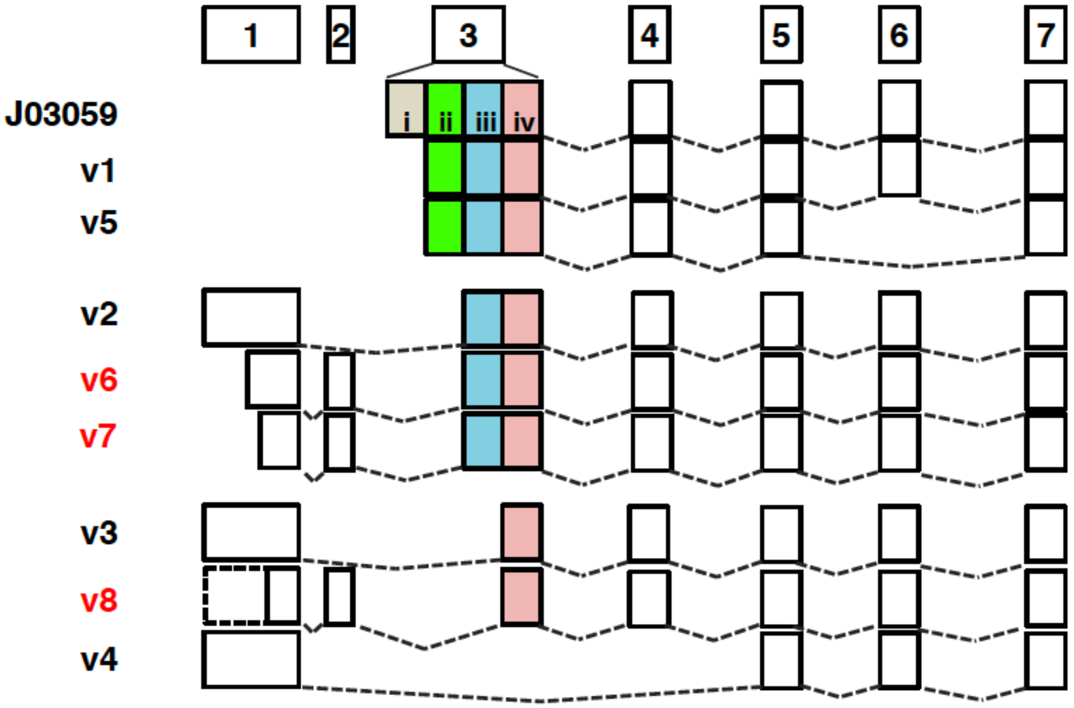


**Supplementary Fig. 1 (continued)**

f. Summary of the exon organization at the 5' ends of the newly identified *GBA* variants. Variant v8 was additionally found by analysis of the PCR products, as shown in (d) and (e). Red, new variants identified by RLM-RACE; blue, additional variant detected by RT-PCR. g. Multiple TSSs and splicing subdomains of *GBA* exon 3. The structural complexity of exon 1 and exon 3 combined with exon 2 among *GBA* variants is shown. The subdomains of exon 3 are shown in different colors based on the different start positions (3i, gray; 3ii, green; 3iii, blue; 3iv, pink). The dotted lines indicate the splicing patterns. For v8, exon 1 was deduced from the results of RLM-RACE and RT-PCR and is indicated with a dotted box.

a

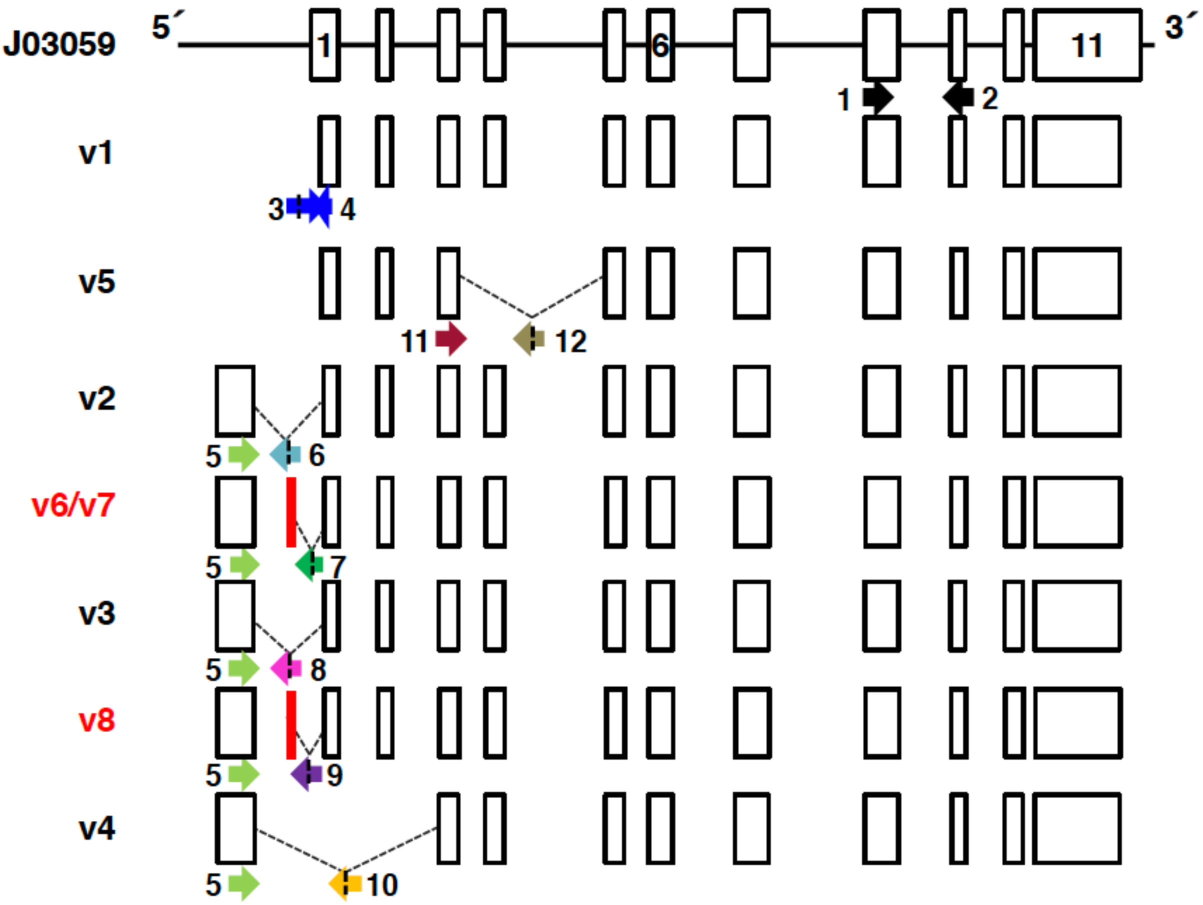


b

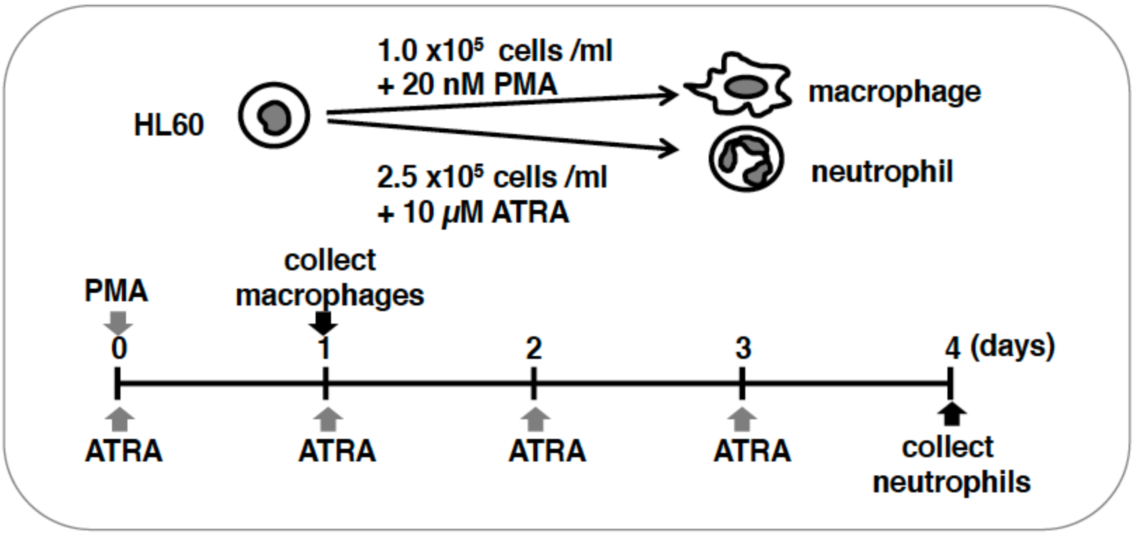


**Supplementary Fig. 2 Cell type-specific expression of *GBA* variants.**

a. Positions of the qPCR primers for the *GBA* variants. The boxes indicate exons, and the colored arrows are the variant-specific primers. The numbers are correlated with Supplementary Table 2. Notes: Primer 3 was designed by combining a partial sequence from the RNA adaptor primer and the 5' end of v1. The numbers on the left side indicate the individual variants. The dotted lines show the splicing events. b. Schematic of macrophage and neutrophil differentiation from HL60 cells. The upper panel indicates the differentiation condition. The lower panel shows a diagram of sample preparation. (continued to next page)

c

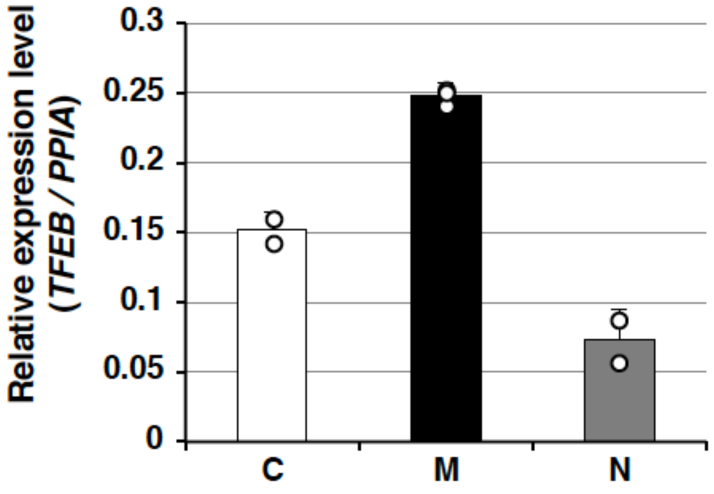


**Supplementary Fig. 2 (continued)**

c. Expression levels of *TFEB* mRNA. The levels of *TFEB* transcripts in induced hematopoietic cells were analyzed by qPCR and normalized to the levels of *PPIA*. Each experiment was performed in triplicate. C, control HL60 cells (no treatment); M, macrophages; N, neutrophils. n=3 biologically independent samples. Data represent the mean ±STDEV.

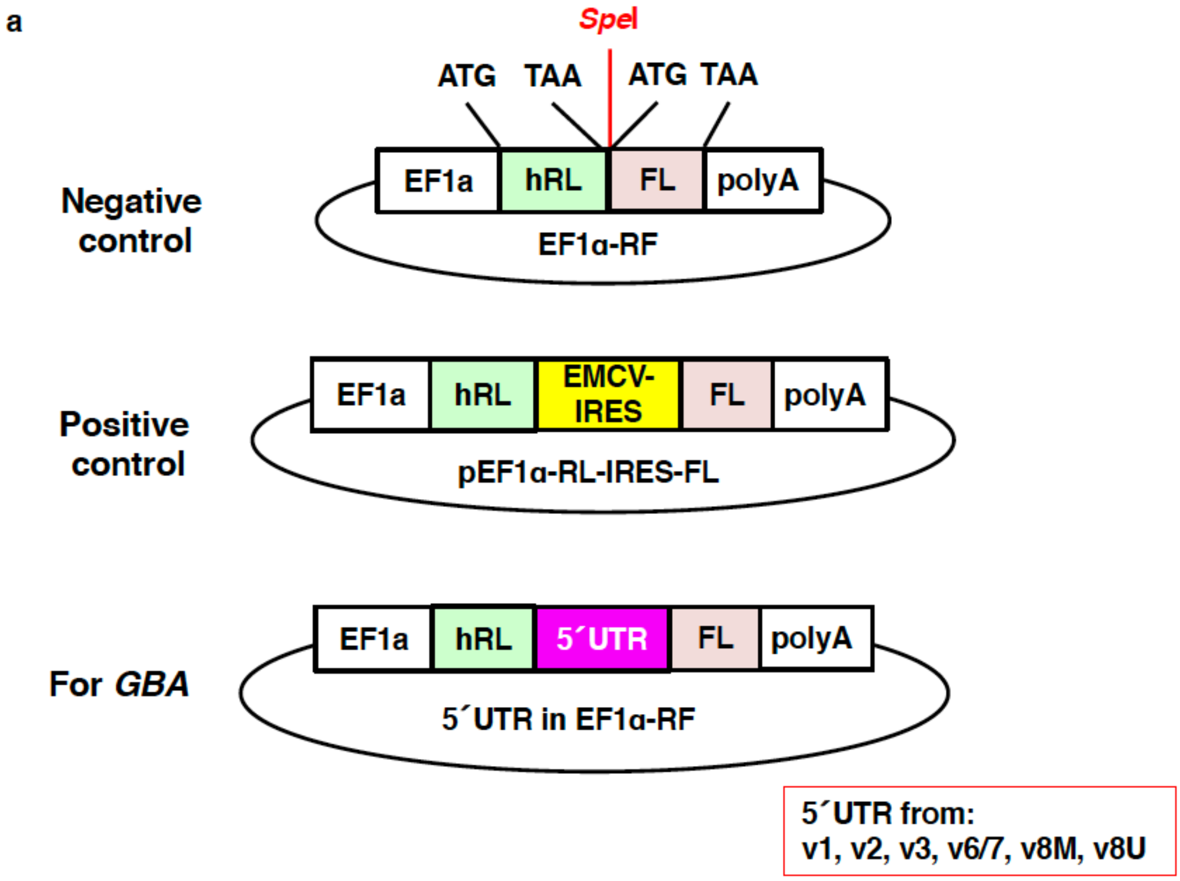


**Supplementary Fig. 3 IRES activity in the 5' UTRs of *GBA* variants.**

a. Reporter constructs for IRES activity. EF1a, EF1α promoter; hRL, Renilla luciferase; FL, firefly luciferase.

(continued to next page)

b

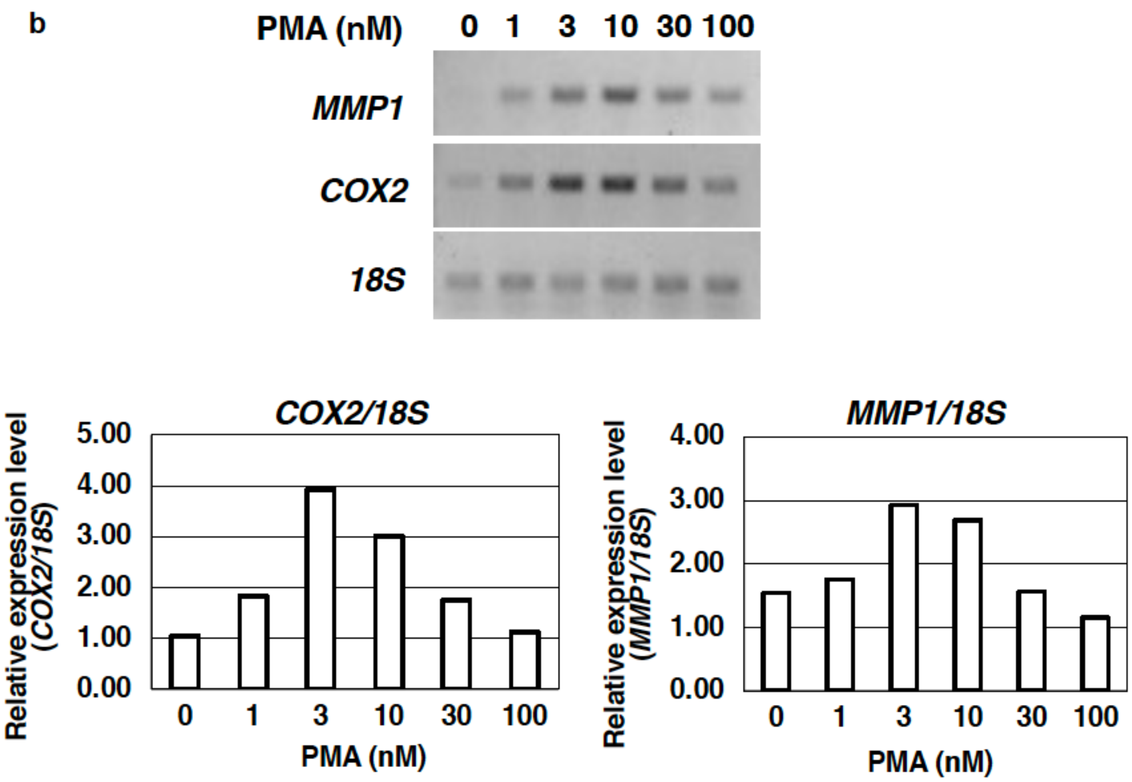


c

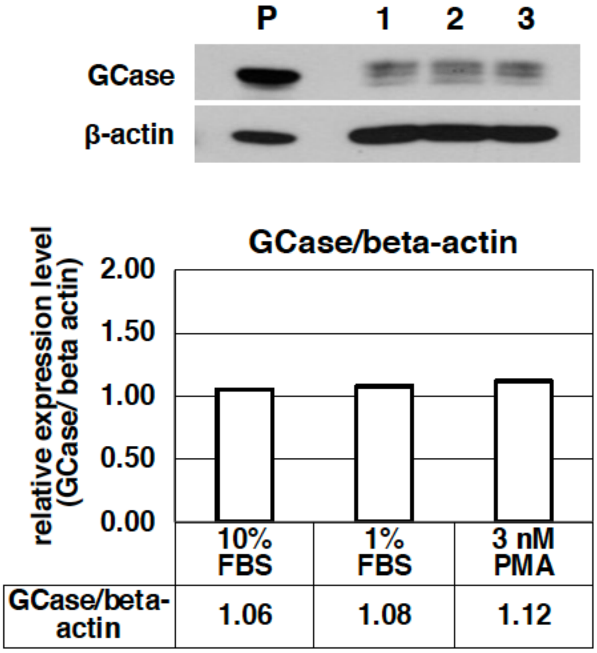


| | 10% FBS | 1% FBS | 3 nM PMA |
|---|---|---|---|
| GCase/beta-actin | 1.06 | 1.08 | 1.12 |

**Supplementary Fig. 3 (continued)**

b. Determination of the effective PMA concentration in OF2 cells. OF2 cells were treated with the indicated concentration of PMA for 24 hours, after which RT-PCR analysis was performed. MMP1 and COX2 are known target genes of PMA signaling in fibroblasts. Each experiment was performed in duplicate and the representative data was shown. The graphs indicate the relative expression level of each gene normalized to 18S ribosomal RNA expression.

c. Effects of PMA on GCase expression and GCase activity in OFs. Each experiment was performed in duplicate and the representative data was shown. P, positive control (expression vector transfected into HEK293 cells); 1, OF2 cells cultured with 10% FBS + D/F12; 2, OF2 cells cultured with 1% FBS + D/F12; 3, OF2 cells cultured with 1% FBS + D/F12 + 3 nM PMA.

## Supplementary Fig. 4 Uncropped blots/gels

**Fig. 1b**

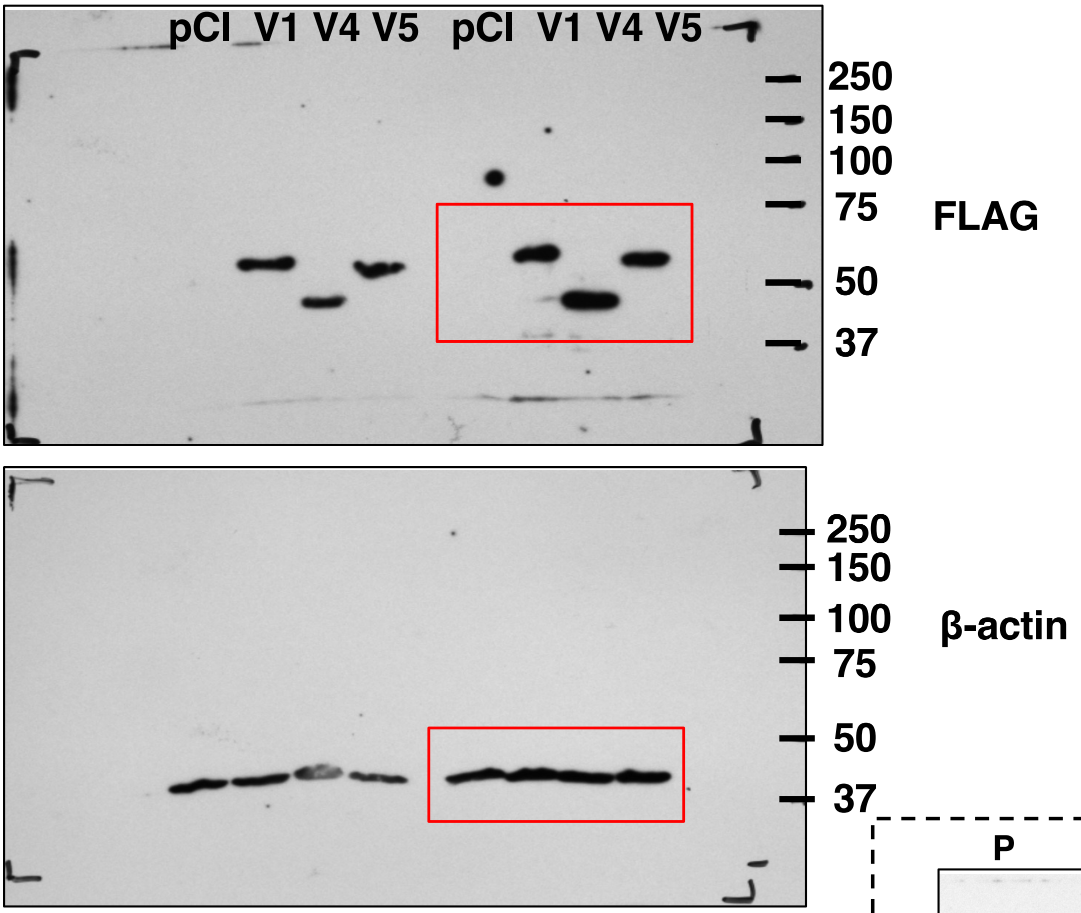


**Fig. 4c**

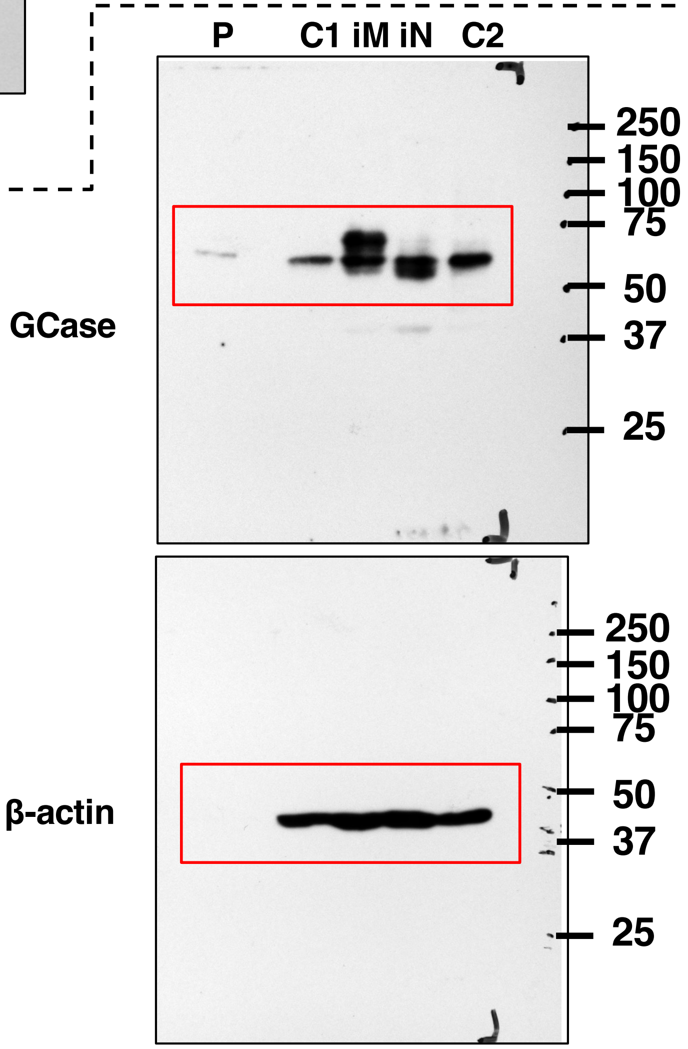

**Supplementary Fig. 4 Uncropped blots/gels**

**Fig. 5c**

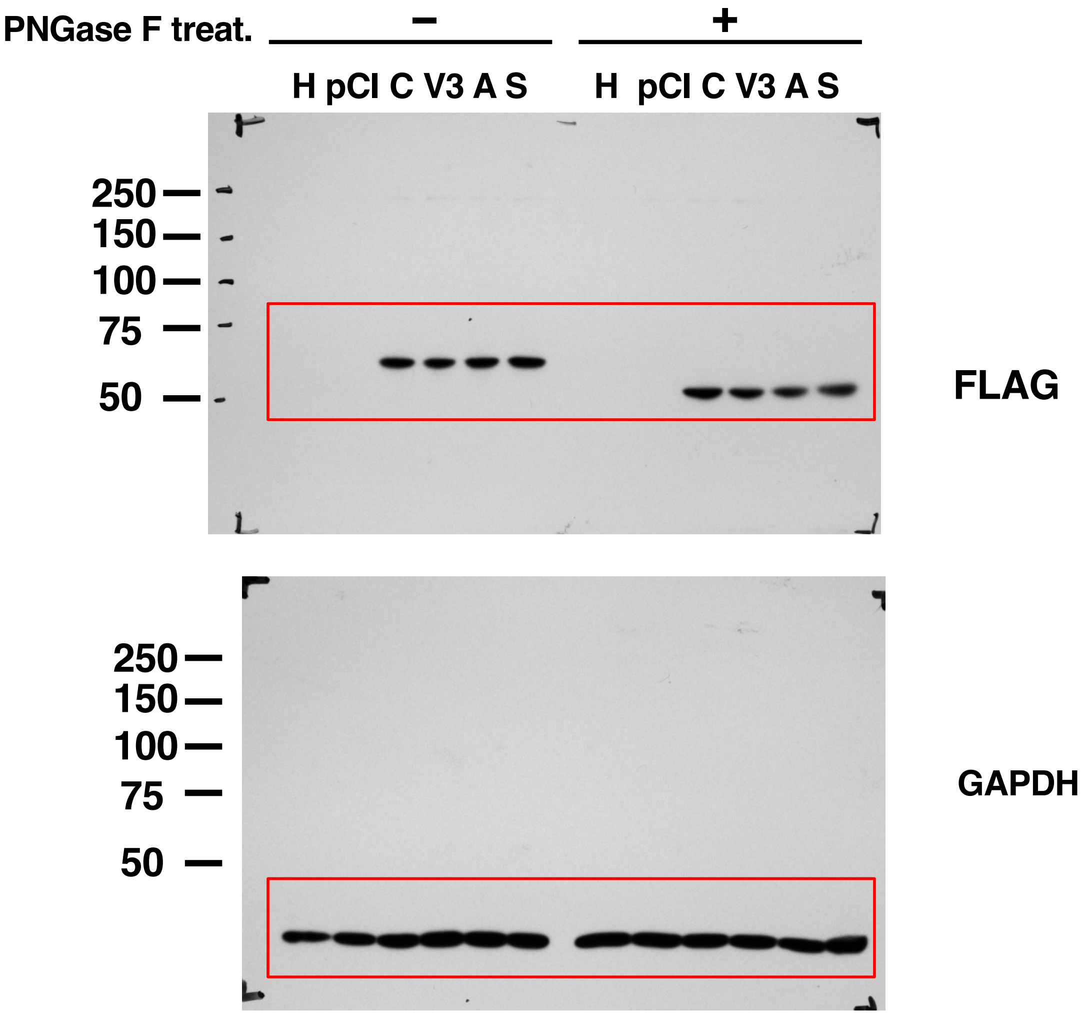

Supplementary Fig. 4  Uncropped blots/gels

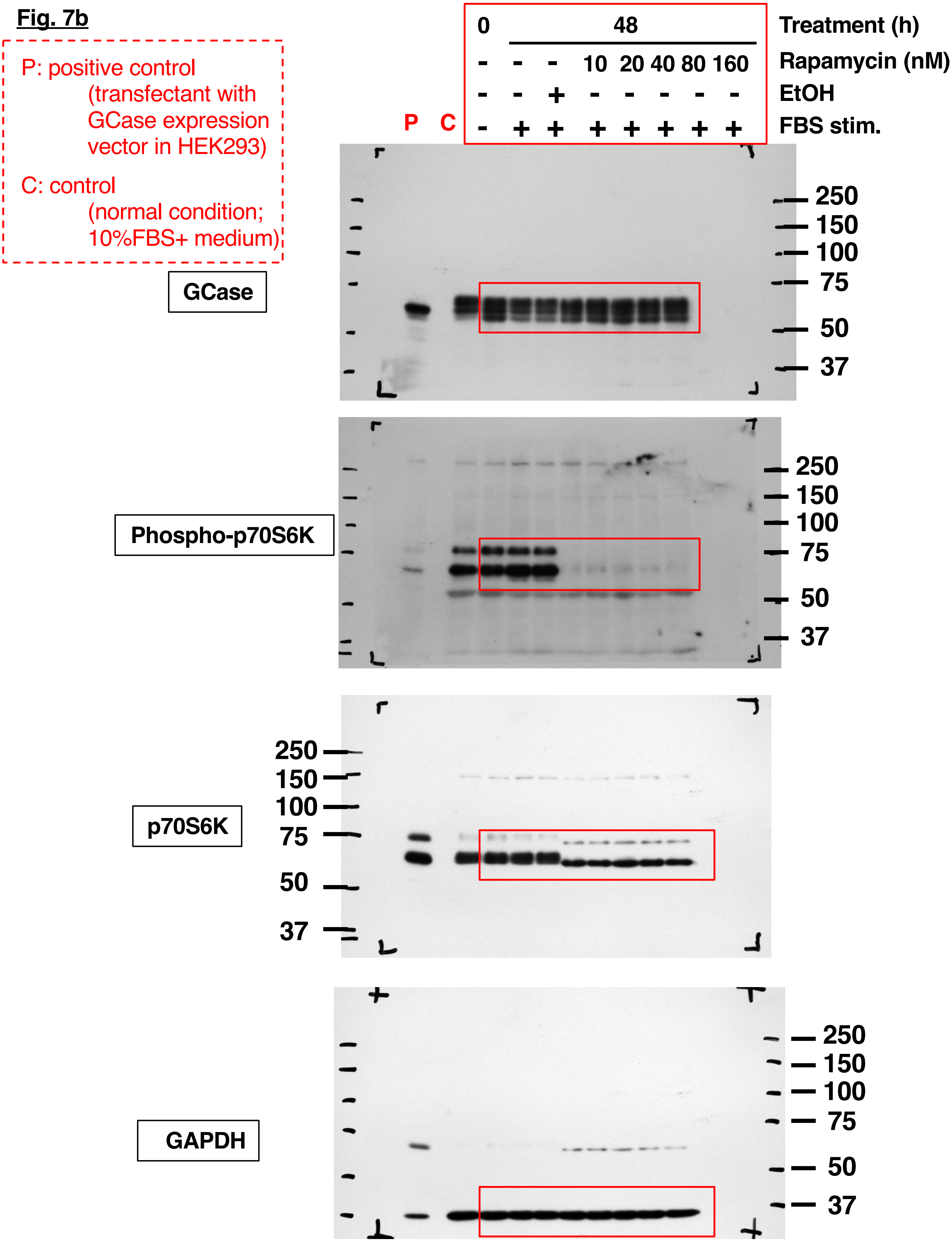

Supplementary Fig. 4  Uncropped blots/gels

<u>Fig. 7c</u>

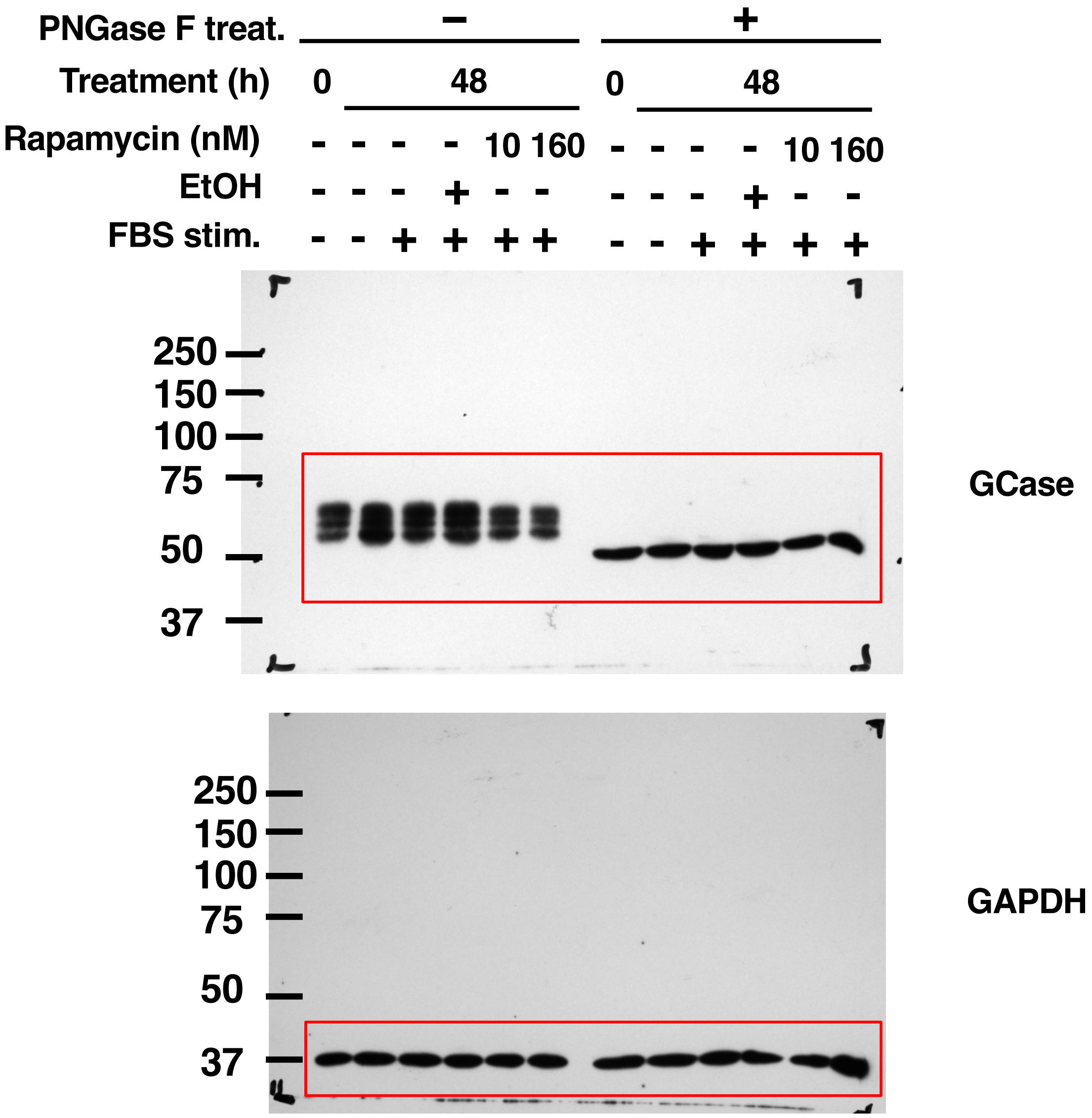

## Supplementary Fig. 4 Uncropped blots/gels

### Supplementary Fig. 1e

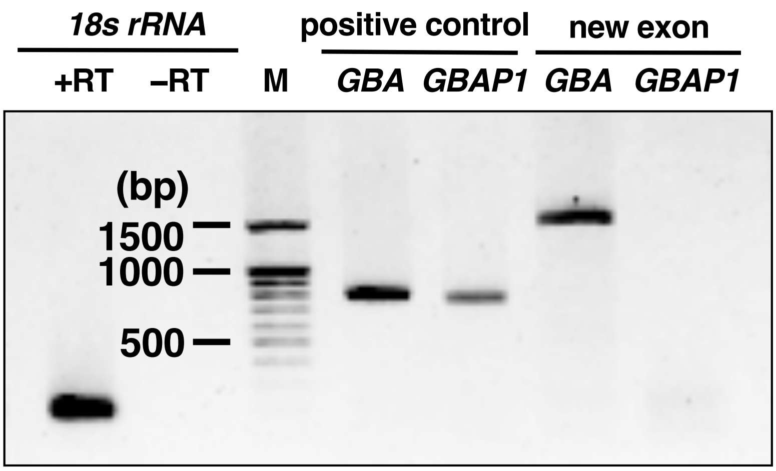


### Supplementary Fig. 3b

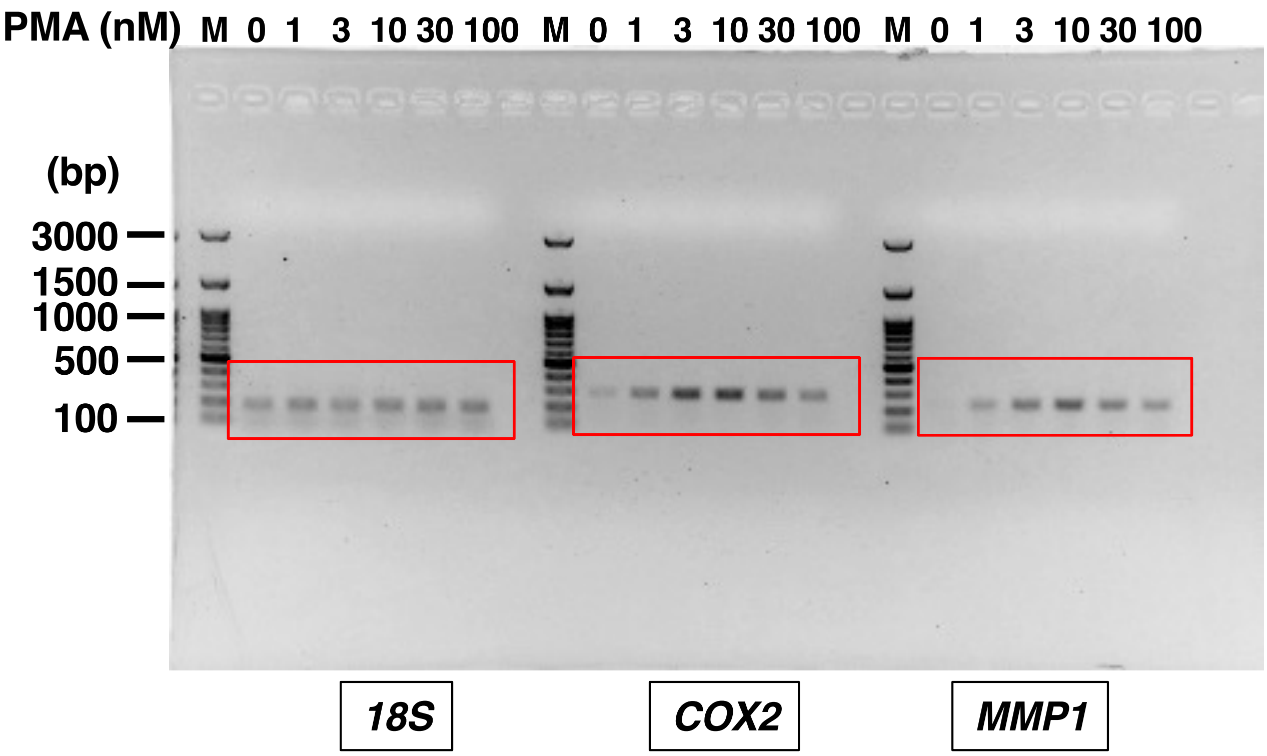

**Supplementary Fig. 4 Uncropped blots/gels**

**Supplementary Fig. 3c**

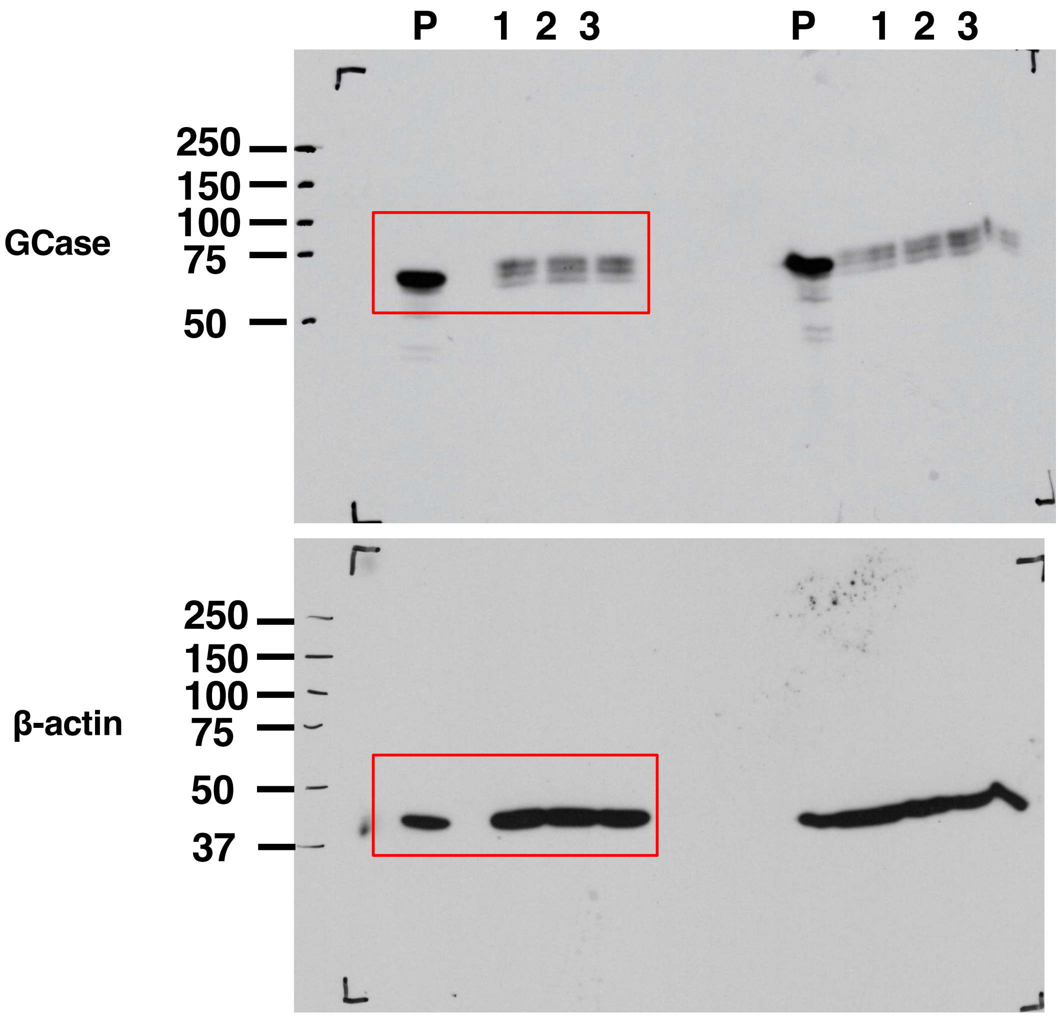


P: positive control
(transfectant with GCase expression vector in HEK293)
1: 10% FBS
2: 1% FBS
3: 3 nM PMA + 1%FBS

**Supplementary Table 1. Gene specific primers for RLM-RACE and new exon confirmation.**

| *Gene specific primers* | Sequence |
|---|---|
| *GBA positive control* | For 5´-CTTCTCCATCCGCACCTACAC-3´<br>Rev 5´-ATTGGGTCCTCCTTCGGGGT-3´ |
| *GBAP1 positive control* | For 5´-CTTCTCCATCCGCACCTACAC-3´<br>Rev 5´-AATGATGGGTTCCAGTCGGTC-3´ |
| *GBA new exon* | For 5´-ATGTTGGTCAGGCTGGTCTC-3´<br>Rev 5´-ATTGGGTCCTCCTTCGGGGT-3´ |
| *GBAP1 new exon* | For 5´-ATGTTGGTCAGGCTGGTCTC-3´<br>Rev 5´-AATGATGGGTTCCAGTCGGTC-3´ |
| *18s rRNA* | For 5´-TACCTGGTTGATCCTGCCAGTAGCAT-3´<br>Rev 5´-CCCGTCGGCATGTATTAGCTCTAGAA-3´ |

cDNA was synthesized using random primers with an RNA-PCR vol. 3.1 kit (Takara Bio) using 500 ng of DNase I (Invitrogen)-treated total RNA derived from DF2 cells. PCR was performed using PrimeSTAR® GXL DNA Polymerase (Takara Bio) with 40 cycles of denaturing at 98°C for 10 sec and annealing/extension at 68°C for 3 min. For the positive control, PCR was performed using GoTaq® DNA Polymerase (Promega) under the following conditions: 1 cycle of denaturing at 95°C for 4 min; 30 cycles of denaturing at 95°C for 30 sec, annealing at 58°C for 30 sec, and extension at 72°C for 2 min; and 1 cycle of extension at 72°C for 7 min. The RT reaction was confirmed by *18S ribosomal RNA* (*rRNA*) expression analysis using GoTaq® DNA Polymerase (Promega), under the following conditions: 1 cycle of denaturing at 95 °C for 4 min; 17 cycles of denaturing at 95°C for 30 sec, annealing at 58°C for 30 sec, and extension for 72°C for 30 sec; and 1 cycle of extension at 72°C for 7 min.

**Supplementary Table 2. Gene specific primers for quantitative PCR.**

| *Gene specific primers* | **Sequence** |
|---|---|
| *1. GBA_specific qPCR F1* | **5´-TCCTGGGATCGAGGGATGCAG-3´** |
| *2. GBA ex8-9R* | **5´-ATTGGGTCCTCCTTCGGGGT-3´** |
| *3. GBA_v1 qPCR F2* | **5´-*AAGGAGTAGAAA*ATCACATGACCC-3´** |
| *4. GBA_v1 qPCR R1* | **5´-TGAAAACTCCATCCCCTCAGGG-3´** |
| *5. GBA_v2-4/v6-8_qPCR F1* | **5´-TGCCTTCAGAGTCTTACTGCGCG-3´** |
| *6. GBA_v2 qPCR R1* | **5´-ATAGAGGATCCACGTCGGCGAAG-3´** |
| *7. GBA_v6/v7 qPCR R1* | **5´-GGATCCACGTTAAAAATAATAATGATGG-3´** |
| *8. GBA_v3 qPCR R1* | **5´-TTCCAGAGTCTCGTCGGCGAAG-3´** |
| *9. GBA_v8 qPCR R1* | **5´-AGAGTCTCGTTAAAAATAATAATGATGG-3´** |
| *10. GBA_ v4 qPCR R1* | **5´-GGGCACCGTCGGCGAAGAGAAAC-3´** |
| *11. GBA_v5 qPCR F1* | **5´-CTCGGTGGTGTGTGTCTGCAATG-3´** |
| *12. GBA_v5 qPCR R1* | **5´-TTATATCCGATTCCTGTGCCCGTG-3´** |
| *13. human GAPDH qPCR F1* | **5´-GACCACTTTGTCAAGCTCATTTCC-3´** |
| *14. human GAPDH qPCR R1* | **5´-TGGTGGTCCAGGGGTCTTAC-3´** |
| *15. PPIA-For* | **5´-ATCTGCACTGCCAAGACTGAGTG-3´** |
| *16. PPIA-Rev* | **5´-TTATTCGAGTTGTCCACAGTCAGC-3´** |
| *17. human TFEB-For* | **5´- AGAAGCGAGAGCTCACAGAT-3´** |
| *18. humanTFEB-Rev* | **5´-GTAATCCACAGAGGCCTTGA -3´** |

*Primer 3 were designed by combination of the partial sequences from RNA adaptor primer (Italic) and the 5´end of v1.

**Supplementary Table 3. Conditions for quantitative PCR analysis.**

| *Gene name* | Gene specific primer set* | Set up for quantitative PCR | | PCR Efficiency[1)] (%) |
|---|---|---|---|---|
| | | 1st step | 2nd step | |
| *GBA v1* | 3 & 4 | 95°C: 60sec (1 cycle) | 95°C:15 sec , 60°C:1 min (40 cycles) | 96 |
| *GBA v2* | 5 & 6 | 95°C: 60sec (1 cycle) | 95°C:15 sec , 60°C:1 min (40 cycles) | 100 |
| *GBA v3* | 5 & 8 | 95°C: 60sec (1 cycle) | 95°C:15 sec , 60°C:1 min (40 cycles) | 105 |
| *GBA v4* | 5 & 10 | 95°C: 60sec (1 cycle) | 95°C:15 sec , 60°C:1 min (40 cycles) | 101 |
| *GBA v5* | 11 & 12 | 95°C: 60sec (1 cycle) | 95°C:15 sec , 68°C:1 min (40 cycles) | 112 |
| *GBA v6, v7* | 5 & 7 | 95°C: 60sec (1 cycle) | 95°C:15 sec , 60°C:1 min (40 cycles) | 101 |
| *GBA v8* | 5 & 9 | 95°C: 60sec (1 cycle) | 95°C:15 sec , 60°C:1 min (40 cycles) | 90 |
| *total GBA* | 1 & 2 | 95°C: 60sec (1 cycle) | 95°C:15 sec , 60°C:1 min (40 cycles) | 102 |
| *GAPDH* | 13 & 14 | 95°C: 60sec (1 cycle) | 95°C:15 sec , 60°C:1 min (40 cycles) | 107 |
| *PPIA* | 15 & 16 | 95°C: 60sec (1 cycle) | 95°C:15 sec , 60°C:1 min (50 cycles) | 92 |
| *TFEB* | 17 & 18 | 95°C: 60sec (1 cycle) | 95°C:15 sec , 60°C:1 min (50 cycles) | |

*Primer number is corresponded to Table 2.
[1)]PCR efficiency was calculated as follows *Exp Hematol*. 30(6):503–512,2002

**Supplementary Table 4. Primer sets for *GBA* v3 cloning with various Kozak sequences.**

| Products | Primer name | Sequence |
|---|---|---|
| GBA V3 | GBA-SacII-NheI-strongV3-F | 5´- TCCCCGCGGGCTAGCTCACCATGGGTGACCGTCTCTTTTCC -3´ |
| | GBA-NheI-adequateV3-F | 5´- CTAGCTAGCAGGGGATGAGTGACCGTCTCTTTTCC -3´ |
| | GBA-NheI-V3-5UTR-F<br>GBA-Hind-R | 5´- TCACTAGTGATTAGTGCTAGCCTCTC -3´<br>5´- TAGCCGAAGCTTTTAGGGATGCAGG -3´ |
| GBAcds | hGBA-cds-F1<br>hGBA-cds R1 | 5´- AGGGGATGGAGTTTTCAAGTCCTTCCAG -3´<br>5´- CTCCATCACTGGCGACGCCACAGGTAG -3´ |
| GBAcds +FLAG | hGBA-FLAG-cds R4 | 5´- CTCCATCATTTATCATCGTCATCTTTATAATCCTGGCGACGCCACAGGTAGGTGTGAATGG -3´ |

**Supplementary Table 5. Primer sets for 5´ UTR cloning of *GBA* variants.**

| Products | Primer name | Sequence |
|---|---|---|
| 5´ UTR of GBA v1 | SpeI-GV1-5UTR-F | 5´- GGACTAGTATCACATGACCCATCCACATC -3´ |
| 5´ UTR of GBA v2, v3, v8 | SpeI-GV2-5UTR-F | 5´- GGACTAGTCTCTCTCTCTCGCTCGC -3´ |
| 5´ UTR of GBA v1, v2, v3, v8 | SpeI-GV1and2-5UTR-R | 5´- GGACTAGTCCCCTCAGGGTCATTAGATG -3´ |
| 5´ UTR of GBA v2, v3, v6/7 | SpeI-GV6/7-5UTR80-F | 5´- GGACTAGTCTCTCTCTCTCTCGCTCGCTCTCTCGCTCTCTCGCTCTCTCTCGCTCGCTCTCTCGCTCTCGCTCTCTCTCTCTCTCCGG -3´ |
| | SpeI-GV6/7-5UTR80-R | 5´- GGACTAGTCCCCTCAGGGTCATTAGATGAAGAGAAGACCACAGGGGTTCCAGAGTCTCTGAAGGATAGAGGATCCACGTTAAAAATAATAATG -3´ |

**Supplementary Table 6. Primer sets for miRNA qRT-PCR**

| Target | Primer name | Sequence |
|---|---|---|
| *GBA* mRNA | GBA_ex9_F | 5´- ATTGGGTGCGTAACTTTGTC -3´ |
| | GBA_ex10_R | 5´- TCCAGGTCGTTCTTCTGACT -3´ |
| *GBAP1* mRNA | GBAP1_F | 5´- GGACCGACTGGAACCCAT -3´ |
| | GBAP1_R | 5´- TCCAGGTCGTTCTTCTGACTG -3´ |
| miR22-3p | miR-22-3p_F1 | 5´- GCTGCCAGTTGAAGAACT -3´ |
