# Supplementary material for: Redefining GBA gene structure unveils the ability of Cap-independent, IRES-dependent gene regulation
Source: Commun Biol. 2022 Jul 13;5:639. doi: 10.1038/s42003-022-03577-5 (PMC9279297; doi:10.1038/s42003-022-03577-5)
Supplement: Supplementary file 4 — Reporting Summary [file 42003_2022_3577_MOESM4_ESM.pdf]

nature portfolio

Corresponding author(s): Keiko Miyoshi

Last updated by author(s): May 26, 2022

Reporting Summary

Nature Portfolio wishes to improve the reproducibility of the work that we publish. This form provides structure for consistency and transparency in reporting. For further information on Nature Portfolio policies, see our Editorial Policies and the Editorial Policy Checklist.

Statistics

For all statistical analyses, confirm that the following items are present in the figure legend, table legend, main text, or Methods section.

n/a	Confirmed	
☐	☒	The exact sample size (*n*) for each experimental group/condition, given as a discrete number and unit of measurement
☐	☒	A statement on whether measurements were taken from distinct samples or whether the same sample was measured repeatedly
☐	☒	The statistical test(s) used AND whether they are one- or two-sided<br>*Only common tests should be described solely by name; describe more complex techniques in the Methods section.*
☒	☐	A description of all covariates tested
☐	☒	A description of any assumptions or corrections, such as tests of normality and adjustment for multiple comparisons
☐	☒	A full description of the statistical parameters including central tendency (e.g. means) or other basic estimates (e.g. regression coefficient) AND variation (e.g. standard deviation) or associated estimates of uncertainty (e.g. confidence intervals)
☒	☐	For null hypothesis testing, the test statistic (e.g. *F*, *t*, *r*) with confidence intervals, effect sizes, degrees of freedom and *P* value noted<br>*Give P values as exact values whenever suitable.*
☒	☐	For Bayesian analysis, information on the choice of priors and Markov chain Monte Carlo settings
☒	☐	For hierarchical and complex designs, identification of the appropriate level for tests and full reporting of outcomes
☒	☐	Estimates of effect sizes (e.g. Cohen's *d*, Pearson's *r*), indicating how they were calculated

Our web collection on statistics for biologists contains articles on many of the points above.

Software and code

Policy information about availability of computer code

Data collection	DNA sequencing were performed by ABI 3130 Genetic Analyzer (Applied Biosystems). qPCR data was analyzed by 7300 Real-Time PCR System (Applied Biosystems) and CFX Connect Real-Time System (Bio-Rad). The image capturing and densitometoric analysis of RT-PCR and western blot analysis were determined by Bio-Rad ChemiDoc XRS system (Bio-Rad). GCase activity was analysed by Verioskan Flash spectral scanning multimode reader (Thermo Fisher Scientific). IRES activity was analyzed by Lumat LB 9507 (Berthold technolosies).
Data analysis	DNA sequence data were analyzed by GENETYX SV/RC software version 17.0.1 (GENETYX, Tokyo, Japan). ΔG (free energy) was analyzed by the IRESPred website (http://196.1.114.46:1800/IRESPred/Typer/IRESPred.html. The potential miRNA binding sites were found using miRBase (http://www.mirbase.org/). The calculations were done by Microsoft Excel for Mac version 16.35. and 16.58.

For manuscripts utilizing custom algorithms or software that are central to the research but not yet described in published literature, software must be made available to editors and reviewers. We strongly encourage code deposition in a community repository (e.g. GitHub). See the Nature Portfolio guidelines for submitting code & software for further information.

Data

Policy information about availability of data

All manuscripts must include a data availability statement. This statement should provide the following information, where applicable:

- Accession codes, unique identifiers, or web links for publicly available datasets
- A description of any restrictions on data availability
- For clinical datasets or third party data, please ensure that the statement adheres to our policy

Gene ID 2629; https://www.ncbi.nlm.nih.gov/gene/2629), The partial sequences of human GBA v6, v7, and v8 mRNA have been deposited into GenBank under the accession numbers LC050340, LC050341, and LC050342, respectively.
The other variant accession numbers which we used in this study are as follows: v1, NM_000157; v2, NM_001005741; v3, NM_001005742; v4, NM_001171811; v5, NM_001171812. Other data supporting the findings of this study are available within the paper and its supplementary information files (included uncropped blots/gels; as Supplementary Figure 4), and Supplementary Data.

Human research participants

Policy information about studies involving human research participants and Sex and Gender in Research.

Reporting on sex and gender	Human oral fibroblasts:<br>OF2: Male, 27-year-old, Asian<br>OF3: Male, 31-year-old, Asian<br>OF4: Male, 27-year-old, Asian<br>References: J. Biosci. Bioeng. 110, 345–350 (2010), Biomed Res. Int. 2015, 121575 (2015)
Population characteristics	See above.
Recruitment	Oral fibroblasts were isolated from oral mucosal tissue from the healthy volunteers in Tokushima University.
Ethics oversight	Institutional Research Ethics Committee of Tokushima University (Project No. 708) based on the individual informed consent and written agreement.

Note that full information on the approval of the study protocol must also be provided in the manuscript.

Field-specific reporting

Please select the one below that is the best fit for your research. If you are not sure, read the appropriate sections before making your selection.

☒ Life sciences ☐ Behavioural & social sciences ☐ Ecological, evolutionary & environmental sciences

For a reference copy of the document with all sections, see nature.com/documents/nr-reporting-summary-flat.pdf

Life sciences study design

All studies must disclose on these points even when the disclosure is negative.

Sample size	Sample sizes were chosen based on the available samples from each data set.
Data exclusions	No data was excluded.
Replication	RLM-RACE experiments were reproduced by two persons. The other experiments were repeated in triplicate or duplicate with similar results.
Randomization	The oral fibroblasts were isolated from three healthy volunteers who were randomly selected.<br>HL60 was purchased from RIKEN Cell Bank (Ibaragi, Japan).
Blinding	Blinding is not relevant to this study, because we would like to know the cell-type specificity.

Reporting for specific materials, systems and methods

We require information from authors about some types of materials, experimental systems and methods used in many studies. Here, indicate whether each material, system or method listed is relevant to your study. If you are not sure if a list item applies to your research, read the appropriate section before selecting a response.

Materials & experimental systems

n/a	Involved in the study
☐	☒ Antibodies
☐	☒ Eukaryotic cell lines
☒	☐ Palaeontology and archaeology
☒	☐ Animals and other organisms
☒	☐ Clinical data
☒	☐ Dual use research of concern

Methods

n/a	Involved in the study
☒	☐ ChIP-seq
☒	☐ Flow cytometry
☒	☐ MRI-based neuroimaging

Antibodies

Antibodies used

1) anti-glucocerebrosidase primary antibodies (G4171, Sigma-Aldrich; 2E2, Abnova)
2) Horseradish peroxidase (HRP)-linked anti-rabbit or mouse IgG secondary antibody (GE Healthcare)
3) anti-rabbit or mouse IgG HRP-linjed Antibody(#7074P2, #7076P2,Cell Signaling Technology)
4) anti-β-Actin primary antibody (#clone AC-15, Sigma-Aldrich)
5) monoclonal anti-FLAG® antibody (#clone M2, Sigma-Aldrich)
6) anti phospho-p70S6k (Thr389) antibody (#9205, Cell Signaling Technology)
7) anti p70S6k antibody (#2708, Cell Signaling Technology)
8) anti GAPDH (14C10) antibody (#2118, Cell Signaling Technology)

Validation

All antibodies used in this study were validated by the suppliers. For glucocerebrosidase antibody, we also prepared the positive control; the glucocerebrosidase expression vector tagged with FLAG sequence was transfected into HEK293 cells.

Eukaryotic cell lines

Policy information about cell lines and Sex and Gender in Research

Cell line source(s)

1) Human oral fibroblasts (OF2, OF3, OF4): Prepared by ourselves (J. Biosci. Bioeng. 110, 345–350 (2010), Biomed Res. Int. 2015, 121575 (2015))
2) Human dermal fibroblasts (TIG-110, -111, -114): Purchased from Health Science Research resource bank (Osaka, Japan)
3) HL60: Purchased from RIKEN Cell Bank (Ibaragi, Japan)

Authentication

None of the cell lines used were authenticated.

Mycoplasma contamination

The used cell lines were not determined mycoplasma contamination by PCR test of cultured media and not detected by Hoechst staining.

Commonly misidentified lines
(See ICLAC register)

According to ICLAC version 9, we did not use the commonly misidentified lines in this study.
